# Multifunctional nanoparticles as a tissue adhesive and an injectable marker for image-guided procedures

**DOI:** 10.1038/ncomms15807

**Published:** 2017-07-19

**Authors:** Kwangsoo Shin, Jin Woo Choi, Giho Ko, Seungmin Baik, Dokyoon Kim, Ok Kyu Park, Kyoungbun Lee, Hye Rim Cho, Sang Ihn Han, Soo Hong Lee, Dong Jun Lee, Nohyun Lee, Hyo-Cheol Kim, Taeghwan Hyeon

**Affiliations:** 1 Center for Nanoparticle Research, Institute for Basic Science (IBS), Seoul 08826, Republic of Korea; 2 School of Chemical and Biological Engineering, and Institute of Chemical Processes, Seoul National University, Seoul 08826, Republic of Korea; 3 Department of Radiology, Seoul National University Hospital, Seoul National University College of Medicine, Seoul 03080, Republic of Korea; 4 Department of Pathology, Seoul National University Hospital, Seoul 03080, Republic of Korea; 5 School of Advanced Materials Engineering, Kookmin University, Seoul 02707, Republic of Korea

## Abstract

Tissue adhesives have emerged as an alternative to sutures and staples for wound closure and reconnection of injured tissues after surgery or trauma. Owing to their convenience and effectiveness, these adhesives have received growing attention particularly in minimally invasive procedures. For safe and accurate applications, tissue adhesives should be detectable via clinical imaging modalities and be highly biocompatible for intracorporeal procedures. However, few adhesives meet all these requirements. Herein, we show that biocompatible tantalum oxide/silica core/shell nanoparticles (TSNs) exhibit not only high contrast effects for real-time imaging but also strong adhesive properties. Furthermore, the biocompatible TSNs cause much less cellular toxicity and less inflammation than a clinically used, imageable tissue adhesive (that is, a mixture of cyanoacrylate and Lipiodol). Because of their multifunctional imaging and adhesive property, the TSNs are successfully applied as a hemostatic adhesive for minimally invasive procedures and as an immobilized marker for image-guided procedures.

## Abstract (web summary)

The use of tissue adhesives for the closure of wounds is of growing interest for surgery and trauma management. Here, the authors develop an adhesive that is biocompatible, enables tissue adhesion and also permits real-time imaging due to the presence of contrast agents for multiple modalities.

Tissue adhesives have various advantages over sutures and staples, including a simpler implementation procedure, more immediate sealing, less pain to patients, lower infection rates, and less trauma on operated tissues^1^,^2^,^3^,^4^. Hence, their clinical demands have grown significantly, and consequently, a number of tissue adhesives based on proteins and synthetic polymers including fibrin, gelatin, polyurethanes, and polyethylene glycol have been developed to accommodate various surgical situations^5^,^6^,^7^,^8^,^9^,^10^. The convenience of tissue adhesives is particularly noticeable in minimally invasive procedures and image-guided surgeries because they are very effective for treating a confined region of interest that is inaccessible with other wound closure methods, which can greatly reduce postoperative complications. Since minimally invasive procedures are typically guided by real-time imaging modalities, such as X-ray fluoroscopy, computed tomography (CT), ultrasonography, and fluorescence imaging, it is useful that the applied tissue adhesives are visible with such imaging modalities for accurate and safe applications^11^,^12^,^13^. Moreover, radiotherapy or minimally invasive procedures to soft organs can also be aided by the tissue adhesives with imaging contrast effects for efficient recognition and follow-up of the target areas along the physiological movement of the organ^14^,^15^.

Nanoparticles have been incorporated with tissue adhesives or hydrogels to enhance their mechanical properties, adhesive strength and electrical conductivity and to provide unique functions such as antibacterial effects^4^,^16^,^17^,^18^. Recently, it was reported that the nanoparticle solution itself can act as an adhesive for hydrogels and biological tissues^19^. Owing to its large surface area and the high adsorption energy between biological tissues and the nanoparticle surface, the adhesive property of nanoparticles is sufficient for various applications, such as hemostasis, wound closure, and scaffold-attachments^20^. On the other hand, nanoparticles have also been employed as biomedical imaging probes owing to their unique physical properties and capability of conjugating functional moieties via surface modification^21^,^22^,^23^,^24^,^25^,^26^,^27^,^28^,^29^. They have been used with various molecular imaging modalities for the sensitive diagnosis of lesions^30^,^31^,^32^,^33^,^34^,^35^,^36^,^37^. On the basis of these two different medical applications of nanoparticles, they may be a promising candidate for multimodal image-guided procedures^38^,^39^,^40^. Herein, we design core-shell structured tantalum oxide-silica nanoparticles as tissue adhesives in image-guided surgery.

## Results

### Designed synthesis and characterization of TSNs

**Figure 1:**
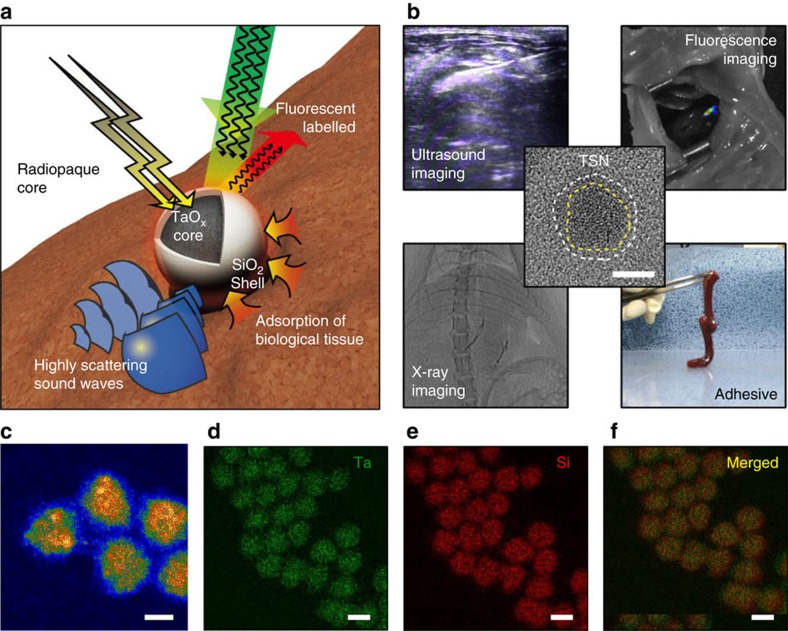
Design and multifunctionality of TSNs. (**a**) Schematic illustration of a multifunctional Tantalum oxide/silica core/shell nanoparticles (TSNs) with a radiopaque core for X-ray imaging, conjugated fluorescent dye for fluorescent imaging, a dense core material with a high sound-scattering effect for ultrasound imaging, and a silica surface for adhesive property. (**b**) Representative images demonstrating multifunctionality of TSNs, and high-resolution transmission electron microscope image of TSN in center. Dashed lines indicate boundaries of core and shell of the nanoparticle. Scale bar indicates 5 nm. (**c**) High angle annular dark field scanning transmission electron microscope image of TSNs. Scale bar indicates 5 nm. (**d**–**f**) Elemental mapping images of tantalum (Ta) (**d**), silicon (Si) (**e**) and their merged image (**f**) obtained by electron energy loss spectroscopy. Scale bar, 10 nm.

We employ silica (SiO_2_) as the shell material to endow the nanoparticles with a tissue adhesive property, because its surface offers high adhesive strength to hydrogels or tissues^19^. Radiopaque tantalum oxide (TaO_x_) is selected as the core material to provide contrast enhancement on X-ray fluoroscopy and CT (Fig. 1a)^41^,^42^,^43^,^44^. TaO_x_ nanoparticles combined with various organic and inorganic moieties have been applied to multimodal imaging and/or theranostics^29^,^45^,^46^,^47^. In the current study, the high density of the TaO_x_ core and the facile modification of the SiO_2_ surface allow for ultrasound (US) and fluorescence imaging.

As designed, the TaO_x_/SiO_2_ core/shell nanoparticle (TSN) glue is clearly visualized by real-time imaging modalities, which facilitates image-guided, less-invasive procedures using this material, and exhibits adhesive property similar to that of a mixture of cyanoacrylate and Lipiodol (CA-Lp) used in clinical practice (Fig. 1b). Cyanoacrylate adhesive, a frequently used tissue adhesive in human, has to be mixed with radiopaque iodized oil (Lipiodol) to be administered under the guidance of fluoroscopy. Moreover, compared to the clinically used CA-Lp, TSNs are highly biocompatible, as evidenced by dramatically reduced cytotoxicity and inflammation reaction. We also demonstrate that TSNs can work as tissue adhesives with the assistance of multimodal imaging modalities in a liver puncture model to stop internal bleeding. Because of their adhesive property and multimodal imaging capability, TSNs are used as an injectable immobilized anatomical marker to aid image-guided surgery.

TSNs were synthesized by the sol-gel reaction of tantalum(V) ethoxide in nanometer-sized microemulsion^41^. After TaO_x_ nanoparticles were generated, the SiO_2_ shell was grown on the TaO_x_ core by sol-gel reaction of tetraethoxysilane (TEOS). High-angle annular dark-field scanning transmission electron microscopy (HAADF-STEM) images show uniform nanoparticles of an overall size of 8.5±0.8 nm with a SiO_2_ shell thickness of 1.3±0.2 nm (Fig. 1c). Shell thickness is easily controllable from 1 to 5 nm by changing the amount of TEOS added (Supplementary Fig. 1). Electron energy loss spectroscopy (EELS) mapping, energy-dispersive X-ray spectroscopy (EDS) mapping, and EDS line scanning show overlapped signals from tantalum and silicon elements, suggesting a core-shell structure (Fig. 1d–f, Supplementary Figs 1 and 2). The core/shell structure is also confirmed by the contrast difference between TaO_x_ and SiO_2_ in the TEM images of TSNs with varying SiO_2_ shell thickness (Supplementary Fig. 3). It is also noteworthy that no separate formation of either silica or tantalum oxide nanoparticles occurred. After the nanoparticles were purified by centrifugation, they were treated with acid to remove any residual adsorbed surfactants that may interfere with the interactions between the nanoparticle surface and biological tissues, leading to the loss of the adhesive property. Removal of the surfactants was confirmed with Fourier transform infrared spectroscopy by the disappearance of peaks related to Igepal CO-520 (Supplementary Fig. 4a). Without the SiO_2_ shell, the reactive TaO_x_ surface causes a severe aggregation of tantalum oxide nanoparticles after the removal of surfactants (Supplementary Fig. 4b), generating micrometer-sized agglomerates (Supplementary Fig. 4b,f). However, the SiO_2_ shell successfully protects TSNs from irreversible agglomeration, resulting in the number-average hydrodynamic diameter of 20.4 nm (Supplementary Fig. 4g). It is crucial that the TSNs should be well dispersed as nanoparticle form because the adhesive property of the nanoparticles is affected by surface adsorption with biological tissue, which will be discussed below. To utilize TSNs for optical image-guided surgery, fluorescent TSNs were prepared by covalent attachment of tetramethylrhodamine isothiocyanate (TRITC) onto the TSN surface. Photoluminescence of the TRITC-conjugated TSNs reveals a typical peak of TRITC at 576 nm (Supplementary Fig. 3l).

### Tissue adhesive property and hemostatic capability

**Figure 2:**
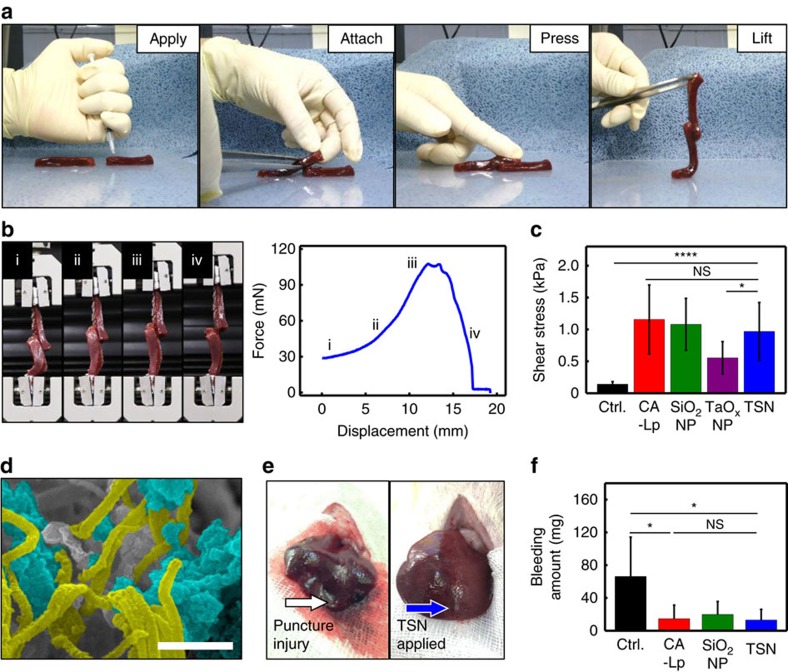
Adhesion strength and hemostatic effect of TSNs in a liver puncture model. (**a**) Photographs showing the lap joint preparation procedure for analysing adhesive strength of various glues on biological tissues of two calf liver ribbons: applying the adhesive, attaching by overlapping the ribbons at the region where the adhesive was applied, gently pressing the ribbon for 30 s, and lifting up and moving the joint to be analysed. (**b**) Photographs showing the lap joint shear test, and a representative force-displacement curve of the lap joint shear test of liver ribbons adhered by TSNs: (i) lifting the lower ribbon, (ii) loading the stress, (iii) ultimate stress point and failure, and (iv) sliding. (**c**) Adhesive shear stress of lap joint attached without glue (control) or with CA-Lp, SiO_2_ NPs, TaO_x_ NPs and TSNs. (Data are shown as mean±s.d., *n*=9, *P<0.05, ****P<0.0001, and NS, not significant *P*>0.05, one-way ANOVA test.) (**d**) Scanning electron microscopy (SEM) image showing the adsorbing and entangling interaction between fibrin fibers and TSNs. TSNs and fibrin fiber are highlighted blue and yellow, respectively. Scale bar indicates 500 nm. (**e**) Photographs of a stab wound after conducting each hemostasis procedure: direct pressure (left), applying TSNs (right). (**f**) Amount of bleeding during each hemostasis procedure. (Data are shown as mean±s.d., *n*=5, *P<0.05, NS, not significant *P*>0.05, one-way ANOVA test.)

To evaluate the suitability of the TSNs for image-guided procedures, their adhesive property was examined with internal tissues containing biological fluids. Liver is an appropriate substrate because it contains abundant biological fluid and blood vessels which have to be sealed inside and it has rather uniform structures among the internal organs. The tissue adhesive property of TSNs was evaluated using a lap joint shear test of liver ribbons. Two liver ribbons were adhered together by gently pressing with a fingertip after spreading 15 μl of the glue solutions (TSNs, SiO_2_ nanoparticles (SiO_2_ NPs), TaO_x_ NPs, CA-Lp, and TSNs coated with Igepal CO-520). Here, CA-Lp was used as a positive control group because it is a U.S. Food and Drug Administration (FDA)-approved tissue adhesive form with a contrast effect and one of the most commonly used glue in image-guided procedures for human. When the upper ribbon was lifted with forceps, it held the lower ribbon without slipping (Fig. 2a). Force-displacement curves were obtained using a universal test machine (UTM) as shown in Fig. 2b. To account for the large deviation of failure forces due to tissue heterogeneity, nine samples were measured for each type of glue (Fig. 2c and Supplementary Fig. 5). The adhesive strengths of all applied adhesives exceeded the intrinsic adhesion strength of biological tissues (Supplementary Fig. 5b).

TSNs exhibit an adhesion strength comparable to that of CA-Lp (1:3) mixture that has enough contrast effect to be visualized in X-ray fluoroscopy (Supplementary Fig. 6). SiO_2_ NP solution also shows similar strength with that of CA-Lp and TSNs. Although the TSNs show much lower adhesion strength than that of commercial CA, the CA is not detectable in X-ray imaging modalities, and unable to be used by itself in image-guided procedures.

To determine the optimal concentration of TSN for adhesive properties, the adhesive strengths were measured with various TSN concentrations. Because the adhesive force increases with increasing nanoparticle concentration (Supplementary Fig. 7), the highest 40 wt% TSN solution was used for hemostasis or markers.

The force-displacement curves of TSNs (Supplementary Fig. 5a) show a similar pattern to that of SiO_2_ NPs, indicating that the adhesion characteristics of TSNs originate from nanoparticle adsorption onto the biological tissue surface and corresponding energy dissipation from the hydrogel-like soft tissues, as previously proposed^19^.

Gluing biological tissues with nanoparticles mainly depends on the ability of nanoparticles to adsorb onto tissues, which involves the interaction between the nanoparticle surface and biological molecules on tissues^19^,^48^. To investigate the adhesive mechanism of TSNs, we prepared various types of TSNs that are surface-functionalized with primary amine, carboxylate, and polyethylene oxide group (Supplementary Table 1 and Supplementary Fig. 8). All of these surface grafted TSNs exhibit no adhesive property (Supplementary Fig. 9). On the other hand, TSNs covered with Igepal CO-520 show fourfold reduced adhesive property than that of bare TSNs that are obtained by the complete removal of Igepal CO-520 by the acid treatment (Supplementary Fig. 9). Meanwhile, agglomeration of TaO_x_ NPs leads to the decrease of adhesion strength by half compared with TSNs (Fig. 2c and Supplementary Fig. 5). These results clearly demonstrate that the silica surface is mainly responsible for the adhesive property of TSNs. Furthermore, silica coating imparts colloidal stability of TSNs.

Adsorption of TSNs was investigated using scanning electron microscopy (SEM) and TEM. SEM and TEM images of the liver samples show localization of TSNs near hepatocytes and leaked subcellular vesicles from the damaged cells by the adsorption of TSNs near lipid layer (Supplementary Fig. 10). Moreover, entangling and adsorption interactions between fibrin fibers and TSNs confirm that the adsorption and spontaneous network formation of TSNs on biological tissues enables the adhesion of cut liver tissues (Fig. 2d and Supplementary Fig. 10).

After confirming their adhesive property, TSNs were applied to hemostasis in a liver puncture model. Since it is difficult to suture a highly vascularized liver wound, either tissue adhesives or sealants are necessary for the warranted closure of hepatic wounds. Puncture injuries were prepared by making a track with a sterile 18-guage needle in the livers of Sprague-Dawley (SD) rats. Subsequently, TSNs, SiO_2_ NPs, or CA-Lp mixture was applied to each track as a hemostatic agent, or direct pressure was put onto the stab wound as a control. Because it is almost impossible to impose pressure in a minimally invasive procedure, no pressure was applied at the sites where the adhesives were treated. As shown in Fig. 2e, both TSNs and CA-Lp successfully stopped bleeding immediately after their application. However, severe blood loss occurred in the control without any adhesives, even though direct pressure was exerted. The amount of bleeding was measured by the weight difference of a filter paper before and after absorbing the blood (Supplementary Fig. 11). A significant decrease in the amount of blood was observed when TSNs, SiO_2_ NPs or CA-Lp was applied, and their hemostatic effects were similar (Fig. 2f).

### Contrast enhancement in various medical modalities

**Figure 3:**
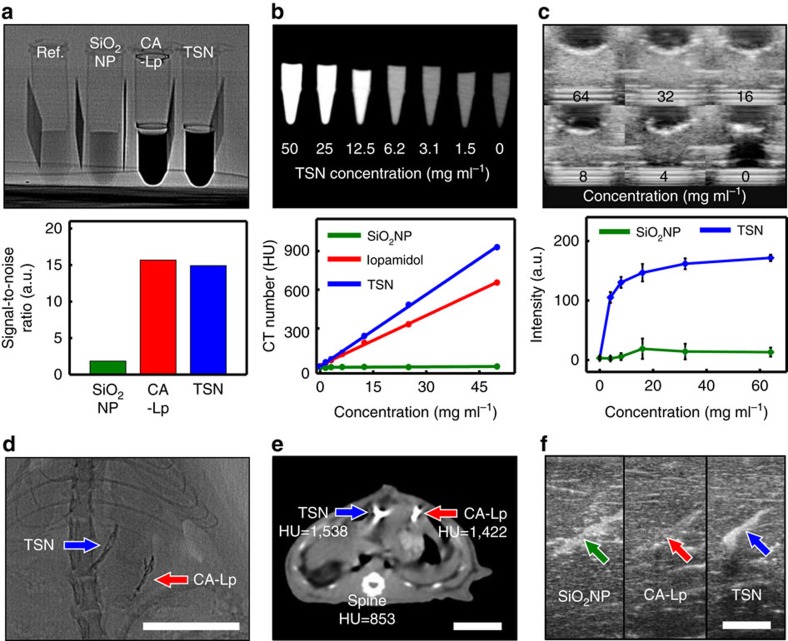
Contrast enhancement of TSNs on various imaging modalities *in vitro* and *in vivo*. (**a**) Contrast enhancement on X-ray fluoroscopy. Fluoroscope image of the tubes containing water (Ref.), commercial silica nanoparticle solution (SiO_2_ NPs, 50 wt%), mixture of cyanoacrylate adhesive and iodized oil (CA-Lp, 75% of Lipiodol, 360 mg ml^−1^ of iodine), and TSNs (40 wt%), and bar plot of the calculated signal to noise ratio from the image). (**b**) Contrast enhancement on computed tomography (CT). CT image of microtubes containing serially diluted TSNs, and line graph of CT contrast enhancement of SiO_2_ NPs, contrast agent (iopamidol), and TSNs against their concentrations. (**c**) Contrast enhancement on ultrasonography. Ultrasonography images of TSN phantoms, and a plot of the mean intensities with s.d. of TSNs and SiO_2_ NPs against their concentrations. (**d**,**e**) *In vivo* images of a Sprague Dawley (SD) rat after application of TSNs and CA-Lp on fluoroscopy (**d**) and CT (**e**). On the CT image (**e**), the mean CT values (Hounsfield unit, HU) of each region of interest (TSN, CA-Lp, and bone) were measured. (**f**) Ultrasonography images of the calf liver where SiO_2_ NPs, CA-Lp and TSNs were injected. Scale bars in **d**–**f** indicate 2 cm.

The contrast effects of TSNs on fluoroscopy, CT and ultrasonography were compared to those of SiO_2_ NPs, CA-Lp, and a commercially available iodine contrast agent (iopamidol). Since X-ray absorption of the contrast agents results in signal attenuation on fluoroscopy, their contrast effects were assessed by signal-to-noise ratios (SNR) (Fig. 3a). The X-ray fluoroscopic image reveals that TSNs significantly attenuate incident X-ray beam, showing a contrast efficiency comparable to that of iodine-containing CA-Lp. The SNR of TSNs is 15 times higher than that of SiO_2_ NPs, demonstrating that the TaO_x_ core is responsible for the high X-ray attenuation^31^,^41^. To evaluate the detection limit of TSNs on CT, we compared the CT numbers (Hounsfield unit, HU) of serially diluted solutions with different TSN concentrations (Supplementary Fig. 12a,d). The contrast effect increases linearly as the concentration increases, and both TSNs and iopamidol are distinguishable from water down to a concentration of 12.5 mg ml^−1^. The contrast efficiencies of TSNs and iopamidol, calculated from the slopes of X-ray fluoroscopy SNR versus concentration, are 0.18 mg^−1^ ml and 0.17 mg^−1^ ml, respectively, while SiO_2_ NPs exhibit a negligible contrast enhancement effect (1.8 × 10^−4^ mg^−1^ ml). The CT contrast enhancement effect of TSNs was also evaluated by the slope of CT number versus concentration and compared with those of iopamidol and SiO_2_ NPs. The contrast enhancement of TSNs is 18.6 HU mg^−1^ ml, which is 1.5 and 150 times higher than those of CA-Lp (12.9 HU mg^−1^ ml) and SiO_2_ NPs (0.12 HU mg^−1^ ml), respectively (Fig. 3b, and Supplementary Fig. 12b,e). The stronger contrast effect of TSNs in CT can be attributed to the higher X-ray photon energy used in CT (applied voltage is 140 kV) compared to that used in fluoroscopy (60 kV), and the well-matched K-edge value of tantalum (67.4 keV) compared with that of iodine (33.2 keV)^31^.

The high mass density of TSNs compared with that of water and flesh offers an effective scattering of US-waves, allowing for the use of TSNs in ultrasonography-guided procedures. The contrast enhancements of TSNs and SiO_2_ NPs were investigated using an agarose phantom loaded with different concentrations of the nanoparticles (Fig. 3c and Supplementary Fig. 12c,f). The US signal of TSNs is distinguishable from the background starting from a nanoparticle concentration of 8 mg ml^−1^, and the signal enhancement of TSNs is three times higher than that of SiO_2_ NPs. It is well known that the back-scattered US generated from Rayleigh scattering is affected by the material size, compressibility and density difference from environment^49^. Therefore, not only a larger hydrodynamic size of TSNs (20.4 nm) than that of SiO_2_ NPs (16.4 nm) but also a high density of the TaO_x_ core (∼8.18 g cm^−3^) contributed to the more intense US signals in comparison to those of SiO_2_ NPs, enabling a much lower detection limit (Supplementary Fig. 4).

The *in vivo* contrast enhancement of TSNs in different imaging modalities was assessed using the aforementioned liver puncture model. As the contrast effects of TSNs and CA-Lp on fluoroscopy are similar, the liver columns filled with each glue exhibit almost identical contrast in the fluoroscope images (Fig. 3d). Notably, the glues are clearly distinguished from the ribs with a similar thickness, which can be advantageous in clinical situations. Similarly, CT imaging reveals that the mean HU value of TSNs is 1.8 times greater than that of the vertebrae and slightly higher than that of CA-Lp (Fig. 3e). Since it is hard to compare directly the contrast enhancements on ultrasonography in a rat due to the small size of its liver, the contrast enhancement of TSNs, CA-Lp, and SiO_2_ NPs was investigated using the calf liver injected with each glue at a depth of 4 cm. The bright mode (B-mode) ultrasonography imaging shows that the signals from SiO_2_ NPs and TSNs are bright enough to be distinguished from the background liver tissue. Although the reflection of US waves from the CA-Lp applied tissue could be detected, its difference from background is insignificant (Fig. 3f).

### Biocompatibility

**Figure 4:**
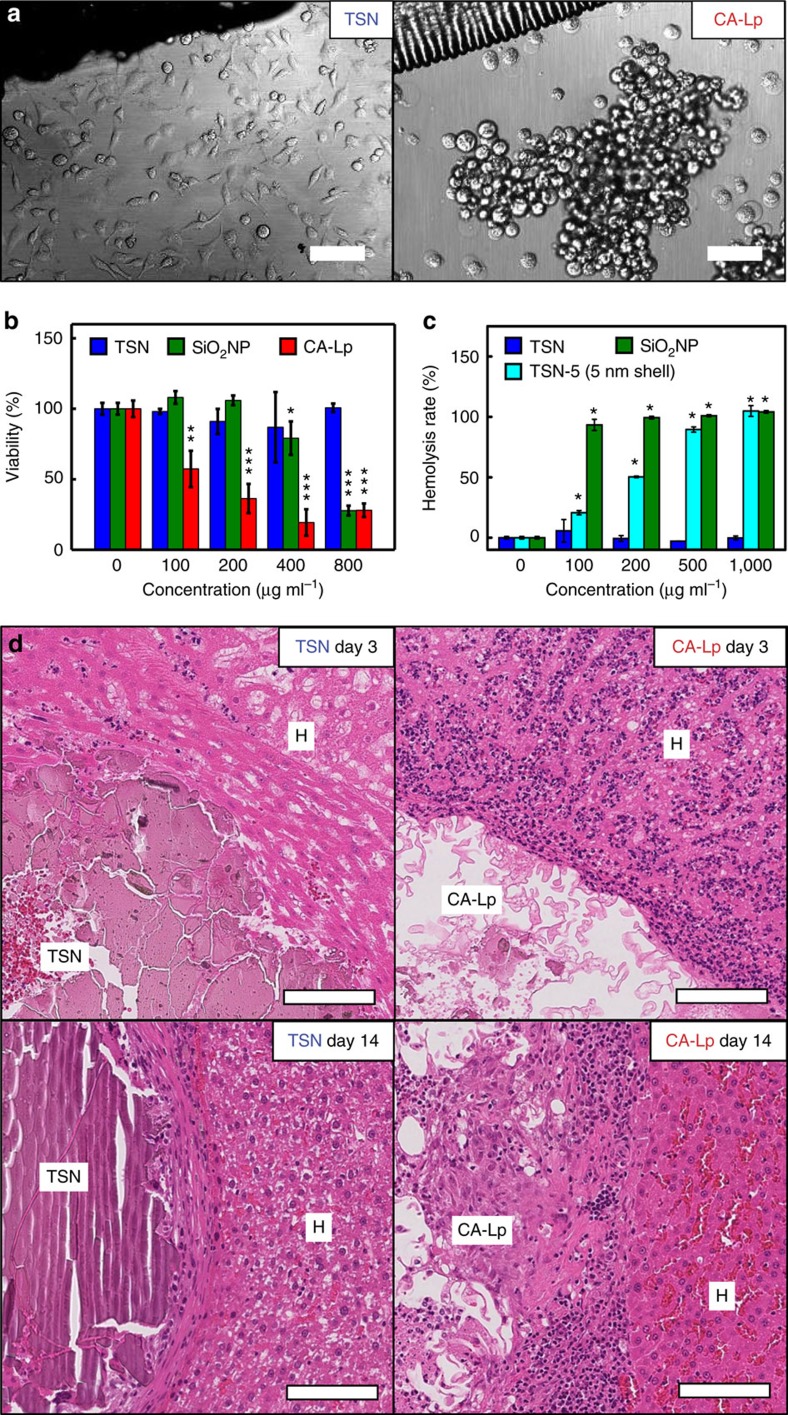
Evaluation of *in vitro* and *in vivo* biocompatibility of TSNs. (**a**) Cellular morphology of mammalian cells (HeLa cell line) incubated with TSNs or CA-Lp. Scale bar indicates 200 μm. (**b**) Viability of cells treated with TSNs, SiO_2_ NPs, and CA-Lp. (Data are shown as mean±s.d., *n*=3, *P<0.05, **P<0.01, ***P<0.001, two-way ANOVA test.) (**c**) Concentration-dependent hemolysis rate of TSNs, SiO_2_ NPs, and TSN-5 with 5 nm-thick silica. (Data are shown as mean±s.d., *n*=3, *P<0.001, two-way ANOVA test.) (**d**) Histological analyses for biocompatibility of CA-Lp and TSN. Rat livers were explanted 3 and 14 days after the hemostasis of a puncture model, and the puncture site and adjacent liver tissue were stained with hematoxylin and eosin (H&E). At both 3 and 14 days after TSN injection, the immune cells (purple dots) were scarcely recruited while numerous immune cells accumulated around the puncture site and in the sinusoids among the hepatocytes (H) at both 3 and 14 days after CA-Lp injection. Scale bar indicates 100 μm.

One of the major hurdles for intracorporeal use of CA-Lp is its severe toxicity and the inflammatory reactions caused by the by-products of CA-Lp such as formaldehyde^5^,^50^,^51^. Recent reports, however, have shown that both SiO_2_ and TaO_x_ nanoparticles are highly biocompatible^23^,^27^,^41^,^52^,^53^. Moreover, tantalum-based stents and implants, and tantalum powder-containing embolic materials (Onyx; Covidien, Irvine, CA) are frequently used in clinics thanks to their biocompatibility^54^. To ensure the biocompatibility of TSNs, their cytotoxicity was evaluated using cell morphology analysis and viability assays. To mimic *in vivo* glue application conditions, a small drop of glue-gel was placed at the bottom center of a culture dish, on which a cell suspension was added. After 24 h, most of the cells incubated with the TSNs were attached to the plate surface regardless of their distance from the gel, while the cells near CA-Lp failed to attach to the plate surface and exhibited a round shape (Fig. 4a). Methylthiazole tetrazolium (MTT) assay shows that the viability of the cells incubated with CA-Lp decreases in a dose-dependent manner while those cells incubated with TSNs exhibit little cytotoxicity up to a concentration of 800 μg ml^−1^, demonstrating excellent biocompatibility of TSNs (Fig. 4b). SiO_2_ NPs have a lower cytotoxicity than CA-Lp, but they still show a significant cytotoxicity at a high concentration of 800 μg ml^−1^. Since SiO_2_ NPs are known to induce hemolysis, the hemolysis effects of TSNs were investigated^55^,^56^. As shown in Fig. 4c, most of the red blood cells were lysed at a SiO_2_ NP concentration of 100 μg ml^−1^, but the hemolysis rate by TSNs was much lower.

Because both TSNs and SiO_2_ NPs have similar silica structure, the observed difference in their hemolytic effect is unexpected. To understand the difference of their hemolysis effect, we evaluated the influence of the nanoparticle size, the amount of fluorescent dye, and the thickness of silica shell on hemolysis rate. Hemolysis rate of silica nanoparticles with sizes of 22, 30, and 105 nm reveals that the largest 105 nm-sized NPs exhibit lower hemolysis rate than that of the 22 nm- and 30 nm-sized silica nanoparticles (Supplementary Fig. 13c), which is consistent with the previous report^55^. The amount of incorporated dye does not affect the hemolysis rate of TSNs and SiO_2_ NPs (Supplementary Fig. 13d–f). Most strikingly, TSN-5 with 5 nm-thick silica shell show much higher hemolysis rate than TSNs with 1 nm-thick silica shell and TaO_x_ NPs without silica shell (Supplementary Fig. 13h).

In the previous reports, hemolysis by silica nanoparticles was attributed to the direct interaction between the cell membrane and silanol groups on the nanoparticle surface, and the mesoporous silica nanoparticles are known to exhibit lower hemolytic effect than dense silica nanoparticles^55^,^56^. The microporous structure of TSNs seems to be responsible for their lower hemolysis rate compared to that of the dense silica nanoparticles (see the gas adsorption data in Supplementary Fig. 14).

*In vivo* biocompatibility of TSNs was assessed by histologically analysing the tissues after applying TSNs or CA-Lp in a liver puncture model (Fig. 4d and Supplementary Fig. 15). A significant difference in the level of tissue inflammation was observed between the biological tissues treated with TSNs and those with CA-Lp. Consistent with the previous reports, an immediate immune reaction was identified around the CA-Lp applied regions within 3 days, and this response developed to a severe inflammation in and around the adhesives at 14 days, and persisted over 56 days^51^. In contrast, the TSN was observed to be innocuous, which is consistent with the previous report on the inertness of tantalum^54^. As the tantalum oxide is a chemically stable material, the degradation of TSNs is slow compared to that of polymer adhesives as shown in histological analysis.

The inertness of the tantalum oxide in TSNs was also supported by the extremely slow dissolution of tantalum in body fluid (Supplementary Fig. 16). Only little portion spreads to other organs including liver, spleen, and lung, and most of the nanoparticles (>99.5%) remains in the applied position (Supplementary Fig. 17). Over six weeks, there was no adverse effect in our blood test, proving their long-term biocompatibility *in vivo* (Supplementary Fig. 18). Considering the intracorporeal use of adhesives for image-guided procedures, their low cellular toxicity, low inflammatory reaction derived from the chemical stability of oxides, and proved long-term biocompatibility can be crucial advantages over CA-Lp.

### Real-time image-guided procedures

**Figure 5:**
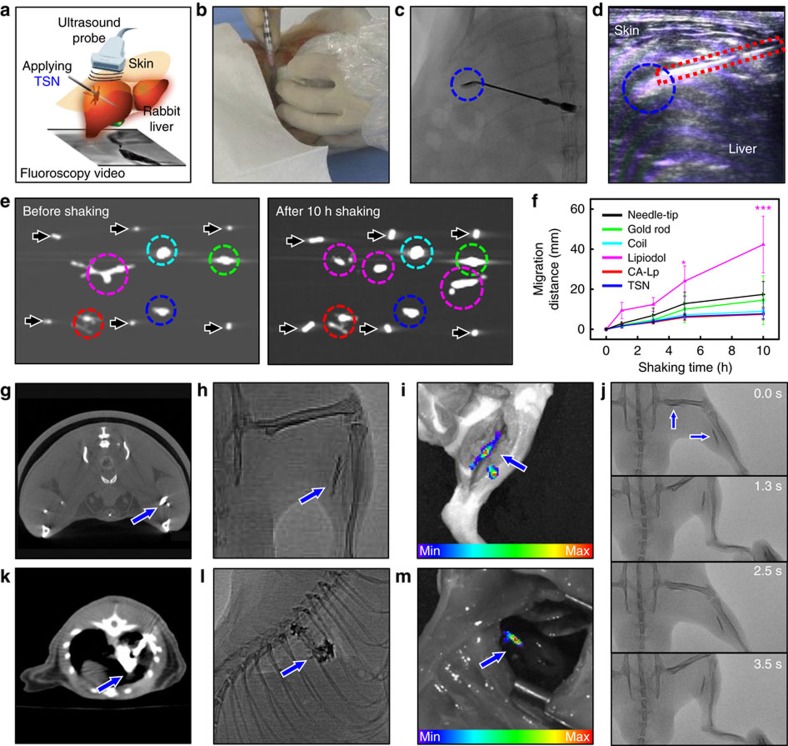
*In vivo* applications of TSNs in multimodal image-guided procedures. (**a**–**d**) TSN adhesive for an incision-free surgical procedure in a rabbit liver puncture model, assisted by simultaneous real-time fluoroscopy and ultrasonography. (**a**) Schematic drawing of the minimally invasive procedures that consist of puncture and closing under two real-time imaging modalities. (**b**) The incision-free image-guided procedure. A photograph showing the sealing process of a liver puncture track by injecting TSNs contained in a syringe. Still images of fluoroscopic video (**c**) and ultrasonography (**d**) during the track sealing with TSNs. Blue dashed circles and red dashed rectangle indicate TSN adhesive and needles, respectively. (**e**,**f**) Mobility evaluation of fiducial markers. (**e**) Top view CT images of the implanted markers before and after 10 h shaking. Black arrows indicate needle tips, and dashed circles indicate the markers, TSNs (blue), gold rod (green), metal coil (cyan), Lipiodol (magenta), and CA-Lp (red) in the liver. (**f**) Average migration distances of the markers in the liver. (Data are shown as mean±s.d. *n*=3, *P<0.05, ***P<0.001 compared to needle-tip, two-way ANOVA test.) (**g**–**j**) Multimodal imaging of the TSNs as an injectable fiducial marker implanted to the calf muscles of a SD rat. (**g**) Transversal micro-CT image showing the location of TSNs near the fibula (calf bone). (**h**) Fluoroscopic image of the implanted TSNs along with the fibula. (**i**) Fluorescent weighted image of the same region. (**j**) A series of still cuts of fluoroscopic video showing that TSN markers follow during extension and flexion movement of leg. (Blue arrows indicate the TSN markers, and the numbers on figure (**j**) represent the time of each frame.) (**k**–**m**) Multimodal imaging of the percutaneously injectable TSN marker in rat lung cancer. (**k**) Axial image of CT. (**l**) Lateral view on fluoroscopy. (**m**) Fluorescence imaging after thoracostomy (Blue arrows indicate the TSNs marked in lung cancer.).

To investigate the potential of TSNs for image-guided surgery, we conducted two surgical demonstrations to show their utility not only as an adhesive for medical intervention with real-time imaging modalities but also as an anatomical marker to guide resection and/or radiotherapy. The liver puncture model was performed in a rabbit without abdominal incision under the guidance of fluoroscopy and ultrasonography (Fig. 5a–d, Supplementary Fig. 19 and Supplementary Movie 1). Using dynamic X-ray fluoroscopic imaging, we could distinguish TSNs from other organs including vertebrae, in spite of low X-ray energy and short exposure time (60 kV and 1 ms, respectively). In ultrasonography, the TSNs that filled the liver track appeared as a bright area. Moreover, there was no bleeding through and on the skin, indicating successful hemostasis of connective tissues and dermal wounds. Thus, TSNs can support minimally invasive real-time procedures with their greatly enhanced contrast effect, enabling both multimodal imaging capability and adhesive property.

In addition to the potential of TSNs as a tissue adhesive, TSNs were also applied as a surgical marker (also called as a fiducial marker) in image-guided procedures. In image-guided procedures or radiotherapy, the movement of soft tissues often hinders localizing and registering an operative region. Some metal-based solid implants, such as a small gold rod or coil, have been used as markers of soft tissues^11^. However, their large physical dimensions usually require complicated insertion procedures that increase the risk of adverse events^57^, and those solid implant markers usually cause streaking artifacts in CT imaging^58^. Although injectable liquid fiducial markers have been proposed, their unwanted migration can lead to serious localization errors as well as complications in distant tissues. To compare mobility, five different fiducial markers including TSNs, gold rod, metal coil, Lipiodol and CA-Lp were implanted in calf lung and liver samples *ex vivo* and these samples were subsequently shaken and CT images are compared. Unlike other materials which generated strong artifacts (gold rod, metal coil) or which moved substantially (Lipiodol), TSNs were immobile during the experiment and caused only negligible artifacts on CT imaging (Fig. 5e,f, and Supplementary Fig. 20). TSNs are ideal fiducial markers due to their detectability by various imaging modalities and their strong adhesion to soft tissues. Moreover, for optical imaging, the fluorescent dye molecules conjugated on TSNs can prevent the rapid dye diffusion that usually results in the blurring of injection sites.

To check the reliability of the TSNs as a fiducial marker within actively moving tissues, TSNs were injected into the thigh and calf muscles of rats. The injected TSNs could be clearly visualized by CT and fluoroscopy, and their positions and shapes were retained during the flexion and extension of the leg (Fig. 5g,h,j and Supplementary Movie 2). Micro-CT images showed that the locations of the TSN markers did not change over two weeks (Supplementary Fig. 21). In addition, TSNs in the muscle were readily detected by fluorescence imaging (Fig. 5i), which can help surgeons recognize a surgical target and perform a safe and accurate operation^59^.

Finally, the feasibility of the TSN marker was evaluated with a rat model of syngeneic lung cancer. Fiducial markers are generally required for successful resection and radiotherapy of lung cancer, but its placement in the lung is challenging because of risks such as migration and distant embolism via the pulmonary veins^60^,^61^. Localizing and marking of target lesions with rhodamine-attached TSNs were conducted with the aid of CT (Supplementary Fig. 22). Neither abnormal breathing nor behaviour was observed during and after the procedures. TSNs were clearly identified in the index tumours on both fluoroscopy and CT (Fig. 5k,l). The opacity of TSNs was high enough to be clearly distinguished from the nearby ribs and vertebrae of the rat. After thoracic incision, fluorescence signals of the rhodamine-attached TSNs were clearly observed from the lesion (Fig. 5m). Both the radiopacity of the TSNs and the high fluorescent signal from rhodamine would help in guiding the resection of the lesion. Considering that the leg and lung move vigorously by the physiological motion of rats, the retention of TSNs represents their great potential as a fiducial marker.

## Discussion

Due to the recent development of medical imaging techniques and interventional devices, many surgeries have been aided by image-guided and minimally-invasive procedures. For example, conventional surgeries for vessel occlusion, bleeding control, internal organ biopsy, spondylosis, and some early-stage tumours (for example, hepatocellular carcinoma) are being replaced by percutaneous angioplasty, catheter-directed embolotherapy, needle biopsy, vertebroplasty and radiofrequency/microwave ablation, respectively, all of which are conducted under the guidance of medical imaging.

However, nanomaterials relevant to the image-guided procedures are rarely suggested for use in medical practice. Ideally, they should be identifiable on clinical imaging (for example, X-ray fluoroscopy, CT, US and magnetic resonance imaging), convenient to apply percutaneously with a catheter or needle, biocompatible to minimize adverse reactions in body, and able to achieve surgical goals (for example, hemostasis, tissue adhesion and tissue marking). Given the unique possibilities of nanomaterials, including high image contrasting effects, adhesive properties, and dispersibility in various fluids, biocompatible NPs have enough potential to replace the conventional materials used in image-guided procedures. In this context, TSNs can be a representative material that satisfies various requirements for medical applications by providing imaging capability, convenient administration, biocompatibility, and effectiveness.

The inertness of tantalum oxide not only provides good biocompatibility, but also hinders its biodegradation in body. Unlike typical imaging studies by systemic intravenous administration of contrast agents, image-guided procedures usually require a very small quantity of NPs. Compared with the required amount of tantalum used as an intravenous CT contrast agent (840 mg kg^−1^)^41^, our study utilized less than one fiftieth of tantalum (15 mg kg^−1^), which accounted for only 0.02–0.05 ml. Moreover, almost all TSNs are remained in the target areas without severe adverse reactions, suggesting that the systemic effects of TSNs can be minor, when administered locally with image guidance.

As a fiducial marker before a surgery, poor biodegradability hardly causes a problem because both the index tumour and the marker will be removed all together (*en bloc)*. In radiotherapy, persistent marking of an index tumour facilitates the consistent targeting and follow-up during serial treatments. As a non-degradable tissue adhesive and a fiducial marker, TSNs exhibit substantially ameliorated inflammatory reactions compared with the clinical adhesive mixture, CA-Lp.

In conclusion, we designed and synthesized multifunctional and biocompatible tantalum oxide/silica core/shell nanoparticles (TSNs) composed of radiopaque tantalum oxide core with multimodal imaging capability and silica shell with a strong tissue adhesive property. As designed, the TSN glue is clearly visualized by clinical imaging modalities including X-ray fluoroscopy, CT, and ultrasonography and exhibits adhesive property similar to that of the CA-Lp. Furthermore, the TSNs cause much less cellular toxicity and less inflammation than the clinically used CA-Lp, which is very important for intracorporeal use of adhesives for image-guided procedures. Moreover, we demonstrated the applications of TSNs to hemostatic adhesive for minimally invasive procedures and as immobilized marker for image-guided procedures by combining their multifunctional characteristics and tissue adhesive property. Given the needs of various tissue adhesives in medicine, our results give additional insights on biomedical applications of designed multifunctional nanoparticles, which can be expanded to image-guided procedure and regenerative medicine as well as drug delivery and cancer diagnosis.

## Methods

### Preparation of TSNs

The TSNs were synthesized by the aqueous sol-gel reaction confined in reverse-micelles based on the previous report^41^. Igepal CO-520 (46 g, Sigma-Aldrich), ethanol (5 ml, Samchun, 99.9%), aqueous NaOH solution (5 ml, 150 mM, Samchun) were mixed with cyclohexane (800 ml, Samchun, 99.5%) to form a microemulsion under vigorous stirring. To the resulting transparent solution, tantalum ethoxide (1 ml, 3.85 mmol, Strem, 99.8%) was injected at room temperature, and stirred for 30 min. Subsequently, 28% ammonium hydroxide solution (5 ml, Samchun) and tetraethyl orthosilicate (TEOS, 1 ml, 4.47 mmol, Acros, 98%) were successively added. The mixture was stirred at room temperature for 12 h. After the reaction, the solvents were removed by evaporation at 60 °C, until the solution became white and viscous. The synthesized nanoparticles were separated by centrifugation, and dispersed in ethanol. To remove the adsorbed surfactants, 5 ml of hydrochloric acid (Daejung, 35%) was added, and the nanoparticles were centrifuged and subsequently washed with ethanol twice. The solution was neutralized by washing twice each with 20 ml of phosphate buffer (pH=8.0, 0.1 M) and distilled water. Finally, the solution was concentrated up to 40 wt% and loaded into a 1-ml syringe.

### Rhodamine conjugation and surface modification of the TSNs

The conjugation of TSNs was performed by mixing acid-treated nanoparticles with dye-conjugated silane. Tetramethylrhodamine isothiocyanate (0.44 mg, Sigma-Aldrich, mixed isomers) and (3-aminopropyl)trimethoxysilane (0.18 mg, Sigma-Aldrich, 97%) were dissolved in 0.5 ml of dimethylformamide (Samchun, 99.5%), and the solution was shaken gently for 30 min. The resulting dye solution was added to the nanoparticle solution dispersed in the phosphate buffer (pH=8.0), and kept being shaken over 8 h. The unreacted dye molecules were removed by several centrifugations, and the resulting nanoparticles were dispersed in distilled water. Other surface modification details were summarized in Supplementary Table 1.

### Preparation of CA-Lp and SiO_2_ NPs

CA-Lp was prepared by mixing Histoacryl (B.Braun Surgical, Germany) and Lipiodol Ultra-Fluid (Guerbet, France) with a volumetric ratio of 1:3, to have a comparable fluoroscope contrast effect, with the iodine concentration of 360 mg ml^−1^. CA-Lp was prepared right before the experiments to avoid the loss of the adhesive properties. To make a comparison with the reported nano-bridging SiO_2_ NPs, Ludox TM-50 (50 wt% of silica) was purchased from Sigma-Aldrich and used without any treatment^19^,^20^.

### Characterization of TSNs and SiO_2_ NPs

TSNs and SiO_2_ NPs were characterized using a TEM (200 kV, JEOL-2,100, JEOL Ltd., Japan) equipped with an electron energy loss spectroscopy mapping (EELS, Quantum 963, Gatan, Inc., USA) and energy-dispersive X-ray spectroscopy (EDS, X-MAX 80T, Oxford). High-resolution HAADF-STEM images were obtained with Cs-corrected STEM (200 kV, JEM-ARM200F, JEOL Ltd., Japan) of National center for inter-university research facilities, Seoul National University. For EDS analysis, due to energy overlap between Si Kα and Ta Mα (Si Kα=1.739 eV, Ta Mα=1.709 eV), the energy range for Ta signal was set to 9.15–9.80 eV to include Ta Lβ_1_ (9.343 eV) and Lβ_2_ (9.651 eV). Fourier transform infrared (FT-IR) spectra were obtained with a JASCO FT/IR-200 (Jasco, Inc., Japan). Hydrodynamic diameters and ζ-potentials of the nanoparticles dispersed in water were analysed with a size analyser (Nanozs, Malvern, Germany). The absorption and emission spectra of the rhodamine-conjugated nanoparticles and free dye were measured with a SpectroV-550 (Jasco Inc.) and a FP-5,500 (Jasco Inc.). Nitrogen adsorption/desorption isotherm was measured by a 3FLEX surface characterization analyzer (Micromeritics, USA).

### Evaluation of contrast effect

Each type of the adhesive (TSNs, SiO_2_ NPs and CA-Lp) was loaded into a 2-ml tube. The contrast effect on fluoroscopy was measured with images acquired by an Allura Xper FD20 (Philips, Netherlands, at 80 kV of tube voltage). The SNR was calculated as SNR=S/N, where S is the mean attenuation of each material and N is the s.d. of background attenuation. Dose-dependent contrast enhancement was examined by comparing the TSNs with SiO_2_ NPs (Ludox TM-50) and a contrast agent (iopamidol, Pamiray, Dongkook pharmaceutical Co., Korea) which were prepared with the same mass concentration. The solutions containing the nanoparticles or the contrast agent were diluted sequentially and dispersed in 1 wt% agarose gel and loaded to 200 μl microtubes. Both fluoroscopy and CT were used to analyse these series of tubes, and the obtained images were evaluated with the OsiriX software (version 4.0; 32 bit; OsiriX foundation Geneva). The clinical CT scanner (Brilliance 64, Philips Medical System, USA) was operated at 140 kVp and 150 mA. To compare the US contrast effect of the TSNs with other glues, 50 μl of each glue was injected to fresh calf liver using an 18-gauge needle while withdrawing a syringe. Afterwards, B-mode US images were obtained with an Accuvix V10 (Samsung Medison co., Ltd, Korea). The mean intensity and s.d. of each region-of-interest were calculated with the ImageJ software (version 1.48v, NIH). To evaluate the contrast effect in serial dilution, the SiO_2_ NPs and TSNs were diluted using 1 wt% agarose, and 200 μl of each solution were cured in a 96-well plate. The plate was immersed in a water bath and the backscattered amplitudes were measured with the same method used in the calf specimens.

### Adhesion strength test

Fresh calf livers purchased from local meat shop (Yuchang Sik-pum, Geumcheon-gu, Seoul, Korea) were cut into several pieces of ribbon shape (45 mm × 10 mm × 4 mm each) with a scalpel and an autopsy blade. Two pieces of the calf liver ribbons were adhered by applying ∼15 μl of each glue. The ribbon pieces were overlapped by 10 mm, and the overlapped portion was gently pressed by a finger for 10 s. To quantify the adhesive strength of the lap joints attached by the glues, lap-shear tests were performed by using an Instron-5,543 electromechanical system (Instron, USA) controlled by the Bluehill software (Ver. 3). The lab joints were placed in a 50 N load cell and the test was performed at a speed of 30 mm min^−1^. Each displacement-force curves were calibrated to the points after slipping, not to be influenced by weights of the lower ribbon of liver.

### Electron microscopic imaging of liver tissue

Electron microscope samples were prepared by fixation and dehydration of the calf liver tissues attached with TSNs. The adhered tissues were fixed with 2.5% of glutaraldehyde and 2% of paraformaldehyde in 0.1 M of sodium cacodylate buffer (pH 7.4), which was then rinsed with cacodylate buffer. Additional fixation was proceeded with 1% osmium tetraoxide solution in cacodylate buffer. After washing the residual fixation reagent with distilled water three times, they were stained overnight with 2% uranyl acetate solution. The dehydration was proceeded gradually with ethanol, by incubating them for 10 min in 30%, 50%, 70%, 80%, 90% ethanol, and finally absolute ethanol three times to eliminate water in the tissues. For SEM sample, the tissues were dried with critical point drier (EM CPD 300, Lieca, Austria) and coated with Pt by sputter coater (EM ACE200, Leica, Austria). For TEM samples, Spurr’s resin was used to ultramicrotome sectioning. Further solvent exchange and infiltration of the resin were proceeded by incubating the dehydrated tissues in propylene oxide twice for 10 min, in 50% Spurr’s resin with propylene oxide for 2 h, and finally in Spurr’s resin overnight. The tissues were embedded in fresh Spurr’s resin at 60 °C for 24 h. The embedded samples were cut with ultramicrotome (EM UC7, Leica, Germany). SEM images were obtained with JSM-6701F (JEOL Ltd.) and energy dispersive X-ray spectrometer (EDS) spectra and element mapping were performed using the attached INCA Energy (Oxford Instruments Analytical Ltd., UK). TEM images were obtained with JEM1010 (JEOL Ltd.).

### Cell culture

HeLa cells (HeLa cell-line, human adenocarcinoma, KCLB-10002) obtained from Korean Cell Line Bank (KCLB, Seoul, Korea) were maintained in Dulbecco’s Modified Eagle’s Medium (DMEM, Gibco). A mycoplasma contamination check was not carried out. The medium was supplemented with 10% fetal bovine serum (FBS, Gibco BRL), 100 μg ml^−1^ streptomycin, and 100 IU penicillin (Gibco BRL). Cells were grown as monolayer cultures in a T75-flask and sub-cultured three times in one week at 37 °C in atmosphere containing 5% CO_2_ and 100% relative humidity.

### Morphological analysis

A 20-μl drop of TSNs or CA-Lp was placed on the middle of coverglass-bottom dish. A Cell suspension in a logarithmic growth phase was added to the dish. Following 24 h of cell seeding onto the dish, bright field images were taken by an inverted confocal microscope (LSM 780, Carl-Zeiss, Germany).

### Viability assay

For the *in vitro* cytotoxicity assay, cells in a logarithmic growth phase were detached and plated (400 μl per well) in 24-well flat-bottom microplates at a density of 40,000 cells per well, which were then left for 24 h at 37 °C to resume exponential growth. After 24 h of recovery, the cellular media were exchanged with the media containing TSNs with various concentrations. As CA polymerizes and hardens with water contact, it is hard to be diluted in PBS or cellular media. Alternatively, we put a drop of CA-Lp gel in the well, letting some molecules or materials be dissolved naturally and affect the cells. After the removal of the media in wells, different volume of CA-Lp (100, 200, 400, and 800 μl) and different concentrations of TSNs were introduced to each culture well, and incubated with 1 ml of cellular media. Following 24 h exposure of the glues to the cells under the same culturing condition, cell viability was assessed by MTT (3-[4,5-dimethylthialzol-2-yl]-2,5-diphenyltetrazolium bromide, Sigma) assay. The assay was performed in quadruplicate in the following manner. The cells were incubated in media with 1 mg ml^−1^ of MTT solution for 2 h. Then the MTT solution was removed and the precipitated violet crystals were dissolved in 1 ml dimethylsulfoxide (DMSO). The absorbance of the solution was measured at 490 nm with microplate reader (Victor X4, Perkin-Elmer, USA).

### Preparation of SiO_2_ NPs and TaO_x_ NPs for hemolysis test

The 105 nm-sized SiO_2_ NPs were synthesized with Stöber process, and 22 nm-sized SiO_2_ NPs were prepared with the same microemulsion method used in the preparation of TSN, except that the precursor was changed to tetramethyl orthosilicate. The 120 nm-sized TaO_x_ NPs were synthesized by aqueous sol-gel reaction of Ta(OEt)_5_ in 90% ethanol aqueous solution.

### Animal study

Ethical approval for all living animal and animal part (for example, isolated tissue, body fluid) experiments was obtained from the Institutional Animal Care and Use Committee (IACUC) of the Clinical Research Institute of Seoul National University Hospital (approval number: 13-0197-C1A0), and the investigators thoroughly followed the IACUC and Association for Assessment and Accreditation of Laboratory Animal Care International guidelines.

### Hemolysis assay

Rabbit blood was collected freshly and stabilized with sodium-heparin. To remove plasma from the whole blood, 4 ml of whole blood was diluted with 12 ml of PBS and centrifuged at 10,000 r.p.m. for 5 min. The supernatant including plasma was removed carefully, and the cells were suspended with additional 12 ml of PBS. Red blood cells (RBC) were isolated by several washing process with 12 ml of PBS and the final solution was diluted with 40 ml of PBS. The nanoparticle solution was diluted with PBS to the various concentrations, and prepared in triplicate. To assess the hemolysis effect, 0.8 ml of the nanoparticle solution and 0.2 ml of the diluted RBC suspension were mixed to reach the final particle concentrations of 100, 200, 500, 1,000 μg ml^−1^. For negative and positive controls, PBS and distilled water were mixed with the same amount of RBC, respectively. The mixtures were incubated at room temperature for 24 h, followed by a centrifugation at 10,000 r.p.m. for 5 min. Each 200 μl of the supernatant of the mixture were conveyed to a 96-well plate and its absorbance was measured at 490 nm with a microplate reader. The percentage of the hemolysis of RBCs was calculated by following formula: percent of hemolysis=((sample absorbance—negative control absorbance)/(positive control absorbance—negative control absorbance)) × 100.

### Degradation experiments of TSN in simulated body fluid

The simulated body fluid (SBF) was prepared as previous report^62^. Each 500 ml of SBF was prepared in polyethylene bottle TSN was immersed with concentration of 200 mg l^−1^, and 400 mg l^−1^. About 10 ml of the sample solutions were extracted each time with centrifugal filter unit (Amicon, 3 kDa). The concentration of free tantalum were determined using inductively coupled plasma mass spectrometry (Elan 6,100, PerkinElmer) of National center for inter-university research facilities, Seoul National University.

### Migration experiments for various fiducial markers

Five different markers such as a contrast agent (Lipiodol), adhesive mixture (CA-Lp), coil, gold rod, and TSNs were implanted or injected into calf liver and lung specimen. The calf liver and lung purchased from local meat shop were prepared freshly by cutting into proper sized cuboid and put into airtight containers. Fifteen needles were attached uprightly to hold the specimens in the containers. Those were imaged with a CT scanner after 1, 3, 5 and 10 h of shaking. The migration distances were measured according to the method of a previous report^63^.

### Liver puncture model in SD rat

Ten male SD rats, at the age of 12 weeks, initially weighing about 400 g, were randomly divided by two groups (that is, five rats per group) and each group was treated by TSNs and CA-Lp. The rats were anesthetized by intramuscular injection of a mixture of zolazepam (5 mg kg^−1^, Zoletil, Virbac, Carros, France) and xylazine (10 mg kg^−1^, Rompun, Bayer-Schering Pharma, Berlin, Germany). To apply the glues to the rat liver and to evaluate their hemostatic effect, the left lobe of the liver of was exposed by a median abdominal incision. After creating a liver stab wound, 18-gauge in diameter (1.27 mm) and 1.5 cm in length, the needle track was closed with TSNs or CA-Lp mixture preloaded within a syringe. 0.03 ml of the adhesives was slowly injected during the withdrawal of the needle. To measure the amount of bleeding, hydrophobic films (Parafilm) were put on each lower and upper side of the liver and filter paper was put right onto the wound area. After bleeding stopped, the amount of the absorbed blood was calculated by measuring the change of the weight of the filter papers.

CT and fluoroscopy images were acquired after the abdominal closure. Fluoroscopic images were obtained with exposure of X-ray at 80 kV for 3 ms. CT imaging parameters of the SD rats were as followed: thickness, 0.6 mm; pitch, 0.648; 120 kVp, 300 mA; field of view, 127 × 127; gantry rotation time, 0.75 s; table speed, 16.7 mm s^−1^.

### Image-guided procedure in a rabbit liver puncture model

Demonstration of image-guided procedures was conducted with a male New Zealand White rabbit weighing 3.5 kg, with the same method in SD rats, except the abdominal incision and exposure of the liver. An 18-gauge stainless steel needle was used to make an intrahepatic track under the guidance of US and fluoroscopy, and 0.05 ml of the adhesives was slowly injected during the withdrawal of the needle. During the image-guided procedure, the operation video and fluoroscopy video were recorded simultaneously. The fluoroscopic images were acquired with exposure of X-ray at 60 kV for 1 ms.

### Percutaneous anatomical marking in rats

Regarding the clinical contexts where fiducial marking for subsequent image-guided surgery or radiotherapy is frequently used (that is, musculoskeletal tumours and lung tumours), experiments were conducted for the leg muscles and syngeneic lung tumour. The muscle and lung marking studies were conducted with two male SD rats (12-week-old, 400 g) and two male F344 rats (12-week-old, 300 g), respectively. Regarding the muscular injection study, 0.02 ml of TSNs was gently implanted into the thigh and calf muscles of male SD rats though a 22-gauge needle. The fiducial markers were observed on micro-CT, real-time fluoroscopy, and fluorescent imaging (IVIS 100, Perkin Elmer). During the fluoroscopic study, the TSN-implanted leg was manually flexed and extended to address whether the fiducial marker was changed in its shape and/or position. To obtain fluorescent signals from the deep muscles, the legs were skinned before fluorescent imaging. For creating a syngeneic lung cancer model, a 0.5 × 0.5 × 0.5 cm sized tumour chip (13762-MAT-B-III cell-line (CRL-1666, ATCC, Manassas, VA, USA)) was injected into the tail vein of male F344 rats. After 10 days, the rats underwent a CT study to identify lung embolization and growth of the tumour chip. A 25-gauge needle was precisely advanced to the lung tumour under CT imaging guidance, and then 0.02 ml of pre-loaded TSNs was slowly injected into the target. The markers were also observed on CT, real-time fluoroscopy, and fluorescent imaging that obtained after thoracostomy.

### Histological analysis

The rats were killed in a CO_2_ chamber, and then the liver was excised and fixed in 10% neutral buffered formalin (10% NBF) for one week. For hematoxylin and eosin (H&E) staining, formalin fixed tissues from each specimen were embedded into paraffin and sectioned into 4 μm thickness. Standard H&E staining was performed to evaluate morphological features of each specimen regarding inflammatory reactions and fibrosis as time passes. Afterwards, all samples were digitalized with optical magnification (× 200) to conduct further analyses. The slides were carefully reviewed by an experienced pathologist who was blinded to the kinds of adhesives.

### *In vivo* toxicity evaluation of TSN

To assess the *in vivo* toxicity of the TSNs, serum chemistry and biodistribution of the NPs were evaluated. 30 μl of the TSNs solution and CA-Lp mixture was applied to SD rats. Since we are interested in two different regions of application, rats were divided into two groups; one is for the liver and the other is for muscle. Rats were killed after 3, 7, 28 and 42 days from the nanoparticle application, and the reticuloendothelial system (liver, spleen and kidney), blood, heart, muscle and lung were collected. In the target organ (liver and muscle), two different regions within the same organ were collected. The tissues were firstly digested with nitric acid, and hydrofluoric acid was added to dissolve TSNs. Ta concentration was determined from inductively coupled plasma mass spectrometer (ICP-MS, NexION300, Perkin Elmer, USA). By collecting 2-ml blood samples from the tail vein of the SD rats, serum biochemistry was examined from the analysis for inflammation (CRP), liver function (ALP, AST and ALT), and kidney function (BUN, CREA), at 3, 7, 21 and 42 days after application of TSN to liver and muscle.

### Statistical analysis

Statistical analyses of intergroup comparisons were performed using one-way ANOVA test with Tukey *post hoc* analysis, or two-way ANOVA test with Bonferroni *post hoc* anaylsis for experiments having two variables. Sizes of sample were determined to empirically for sufficient statistical power. The specific statistical tests and significance level are noted in the figure captions. For calculation of statistical analysis, computational programs including Origin 9.0 and GraphPad Prism 5.0 were used. The investigators were not blinded to group allocations during the experiments and analyses except for the histological analysis, and no randomization method was used.

### Data availability

Data supporting the findings of this study are available within the article and its Supplementary Information Files and from the corresponding author on reasonable request.

## Additional information

**How to cite this article:** Shin, K. *et al*. Multifunctional nanoparticles as a tissue adhesive and an injectable marker for image-guided procedures. *Nat. Commun.* **8,** 15807 doi: 10.1038/ncomms15807 (2017).

**Publisher’s note:** Springer Nature remains neutral with regard to jurisdictional claims in published maps and institutional affiliations.

## Supplementary Material

Supplementary Information Supplementary Figures, Supplementary Table and Supplementary References.

Supplementary Movie 1 Demonstration of image-guided intervention. Image-guided procedure of incision-free liver puncture model of a rabbit. This movie contains operational video, fluoroscopy and ultrasonography. They are simultaneously visualizing the location of the surgical site, needle, and TSN adhesive. To obtain clear images on fluoroscopy, ultrasonographic images were acquired separately, and synchronized with the operation video.

Supplementary Movie 2 Fluoroscopic video of TSN markers in the leg of a SD rat. The markers kept its location and shape in spite of manual flexion and extension of the leg.
