# Supplementary material for: Multifunctional nanoparticles as a tissue adhesive and an injectable marker for image-guided procedures
Source: Nat Commun. 2017 Jul 19;8:15807. doi: 10.1038/ncomms15807 (PMC5524935; doi:10.1038/ncomms15807)
Supplement: Supplementary Information — Supplementary Figures, Supplementary Table and Supplementary References. [file ncomms15807-s1.pdf]

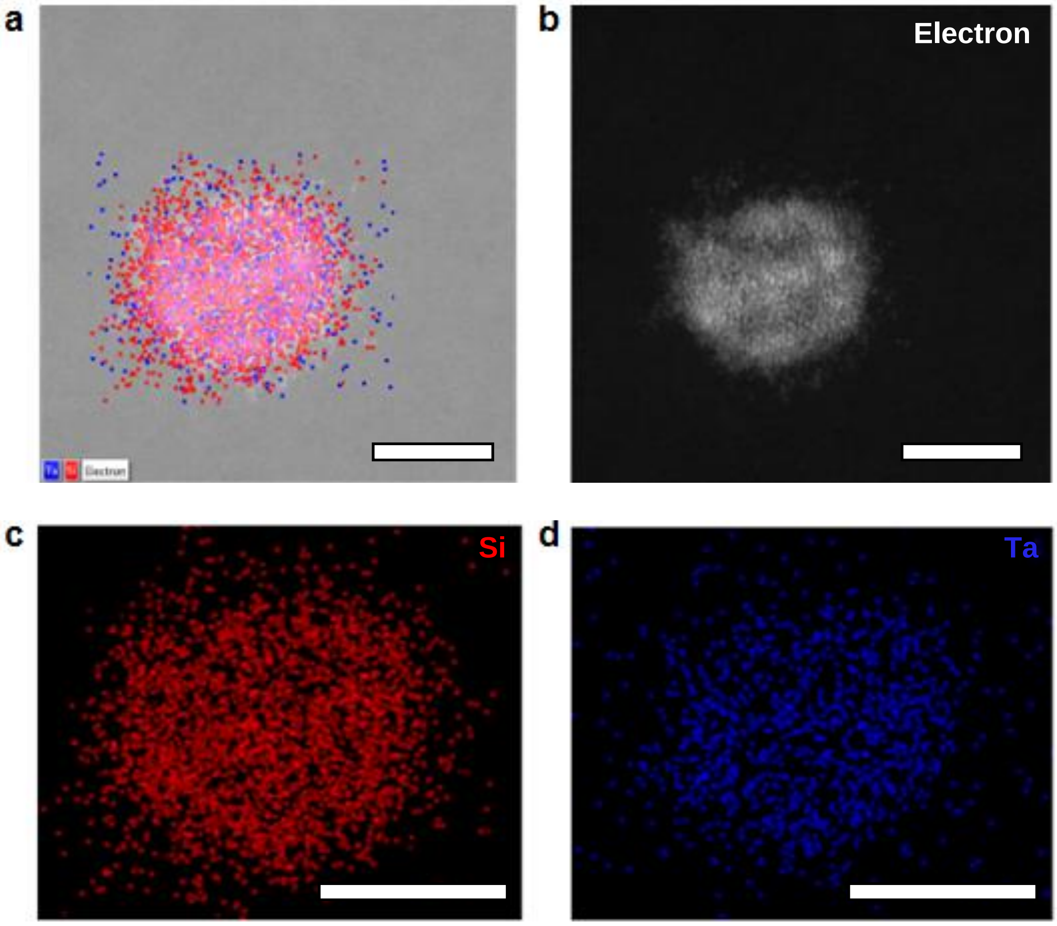

Supplementary Figure 1 | EDS mapping images for TSNs. a, Overlay image of electron, Si and Ta images. **b,** Electron image. **c**, Si mapping image. **d**, Ta mapping image. Scale bars indicate 5 nm.

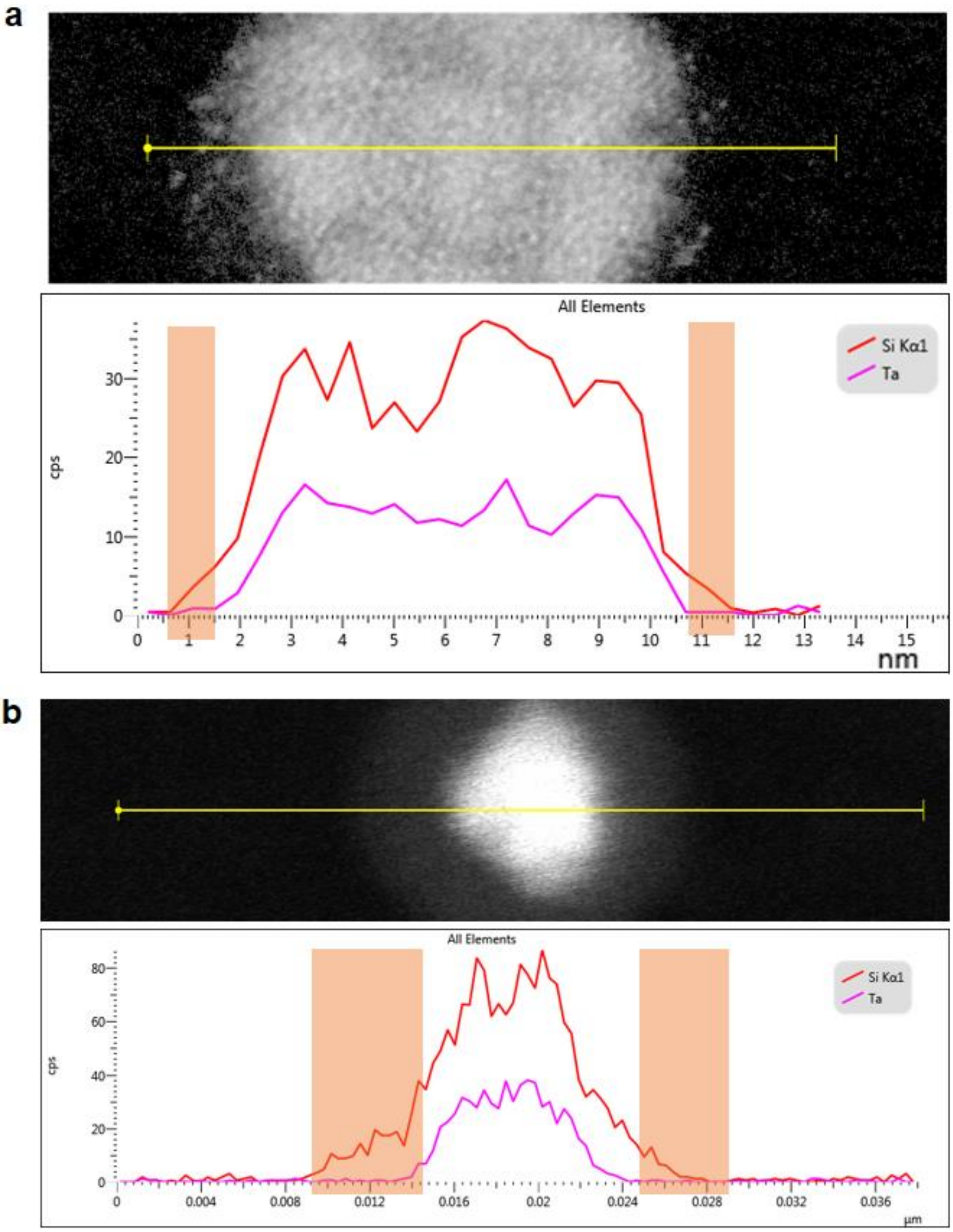

Supplementary Figure 2 | EDS line scanning images and spectra. Line scanning image and spectrum for **a**, TSN (coated with silica layer at the ratio of 1:1). **b**, TaO_x/SiO_2 core/shell NPs coated with silica layer at the ratio of 1:8. Orange color-shaded area shows silica shell region in the spectrum.

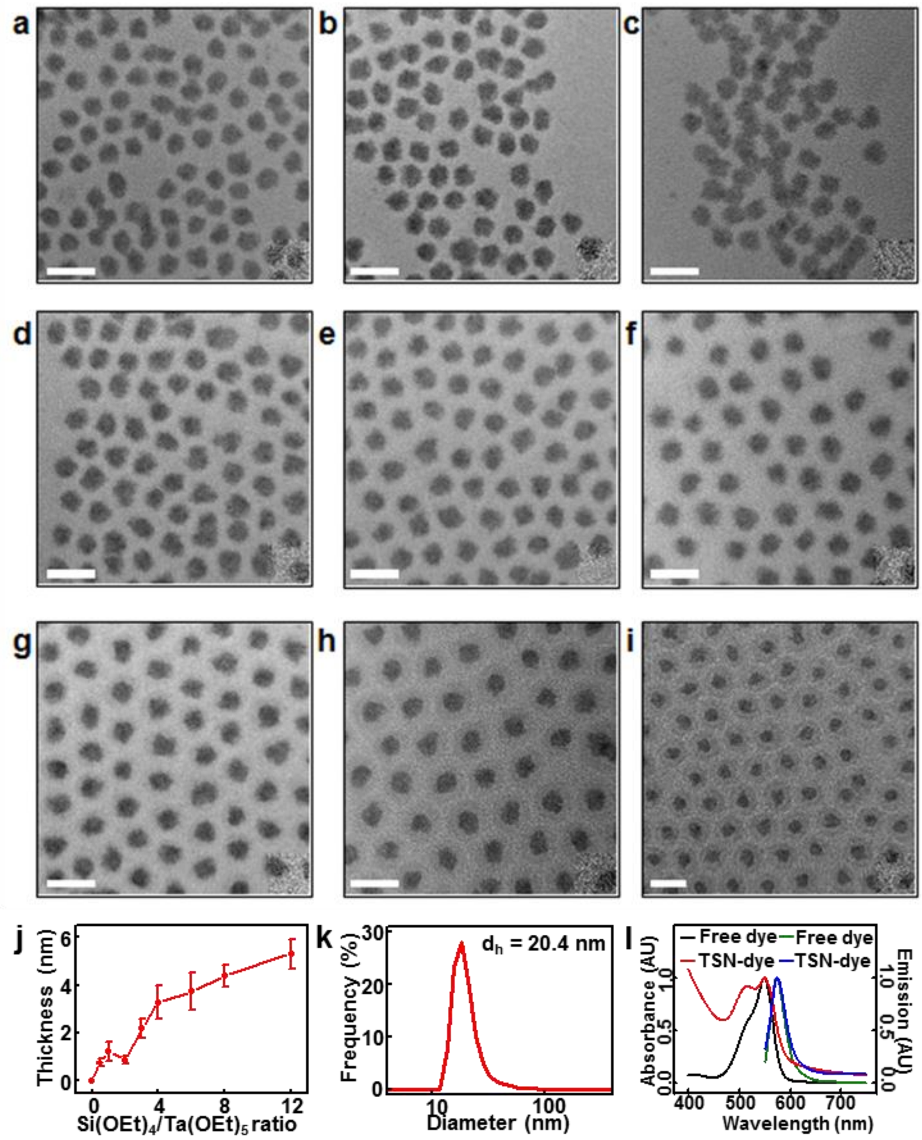

Supplementary Figure 3 | Thickness control and dye attachement of TSNs. a-i, Representative TEM images of the TaO_x/SiO_2 core/shell nanoparticles (TSNs) with different thicknesses of SiO_2 coating from a varied amounts of tetraethyl orthosilicate added. The molar ratios of the amount of silicate precursor over that of tantalum ethoxide precursor are 0 (a), 0.5 (b), 1 (c), 2 (d), 3 (e), 4 (f), 6 (g), 8 (h), 12 (i), respectively. Scale bars are 20 nm. **j**, SiO_2 shell thickness on the molar ratio of silicate precursor added over tantalum precursor. **k**, Number distribution of the hydrodynamic diameter of TSNs analyzed by dynamic light scattering (DLS) **g**, Absorption and emission spectra of the tetramethylrhodamine isothiocyanate (TRITC) molecule and the TRITC-conjugated TSNs.

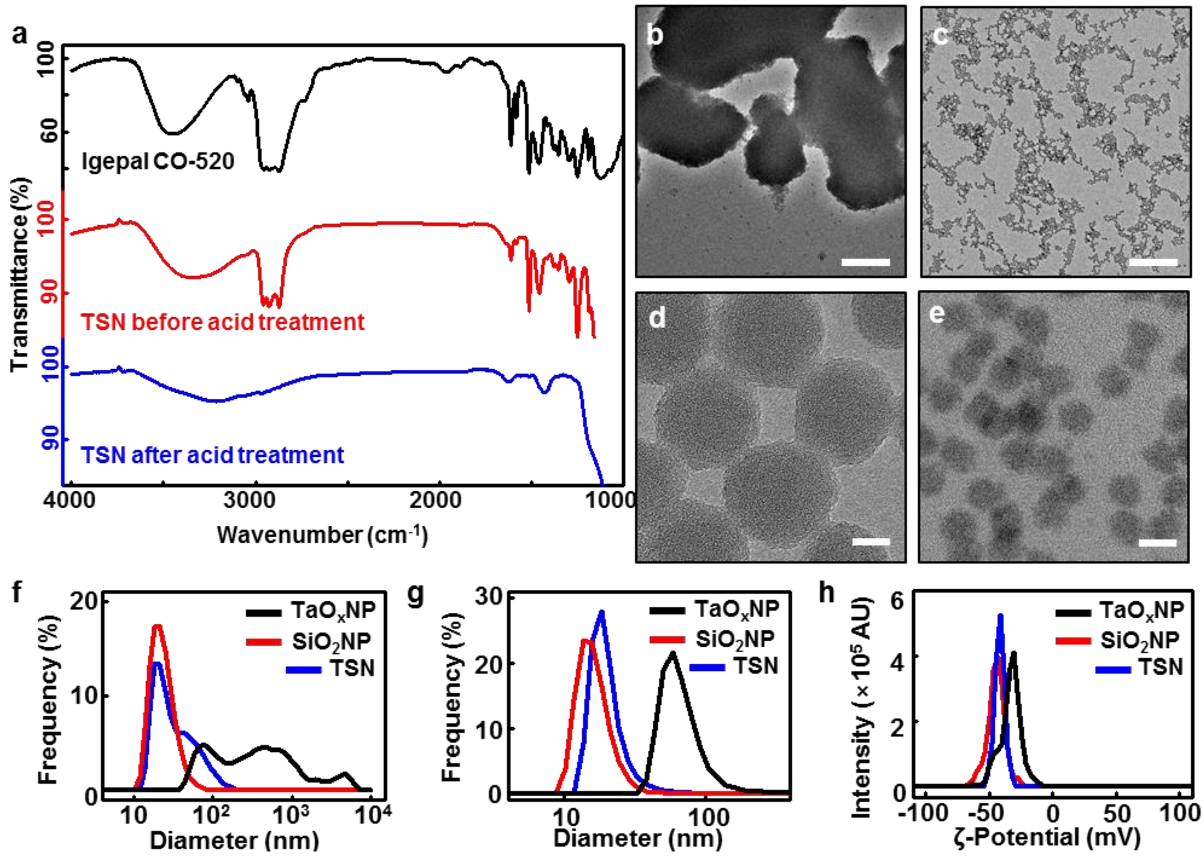

Supplementary Figure 4 | Characterization and comparison of TSNs, SiO_2 NPs, TaO_x NPs. **a,** FT-IR spectra of surfactant, Igepal® CO-520 (black line), TaO_x/SiO_2 core/shell nanoparticles (TSNs) before the acid treatment which the surfactants are adsorbed (red line), and TSNs after the acid treatment (blue line). **b,** TEM image of TaO_x nanoparticles without SiO_2 shell, which is agglomerated to micron-size. (Scale bar indicate 200 nm) **c,** TEM image of TSNs after the acid treatment, which are dispersed well. (Scale bar indicates 200 nm) **d,** high magnified TEM images of SiO_2 NPs. (Scale bar indicates 10 nm) **e,** high magnified TEM images of TSNs. (Scale bar indicates 10 nm) **f,** Volume distributions of hydrodynamic diameter of SiO_2 NPs, and TSNs. **g,** Number distribution. **h,** ζ-potential intensity distributions.

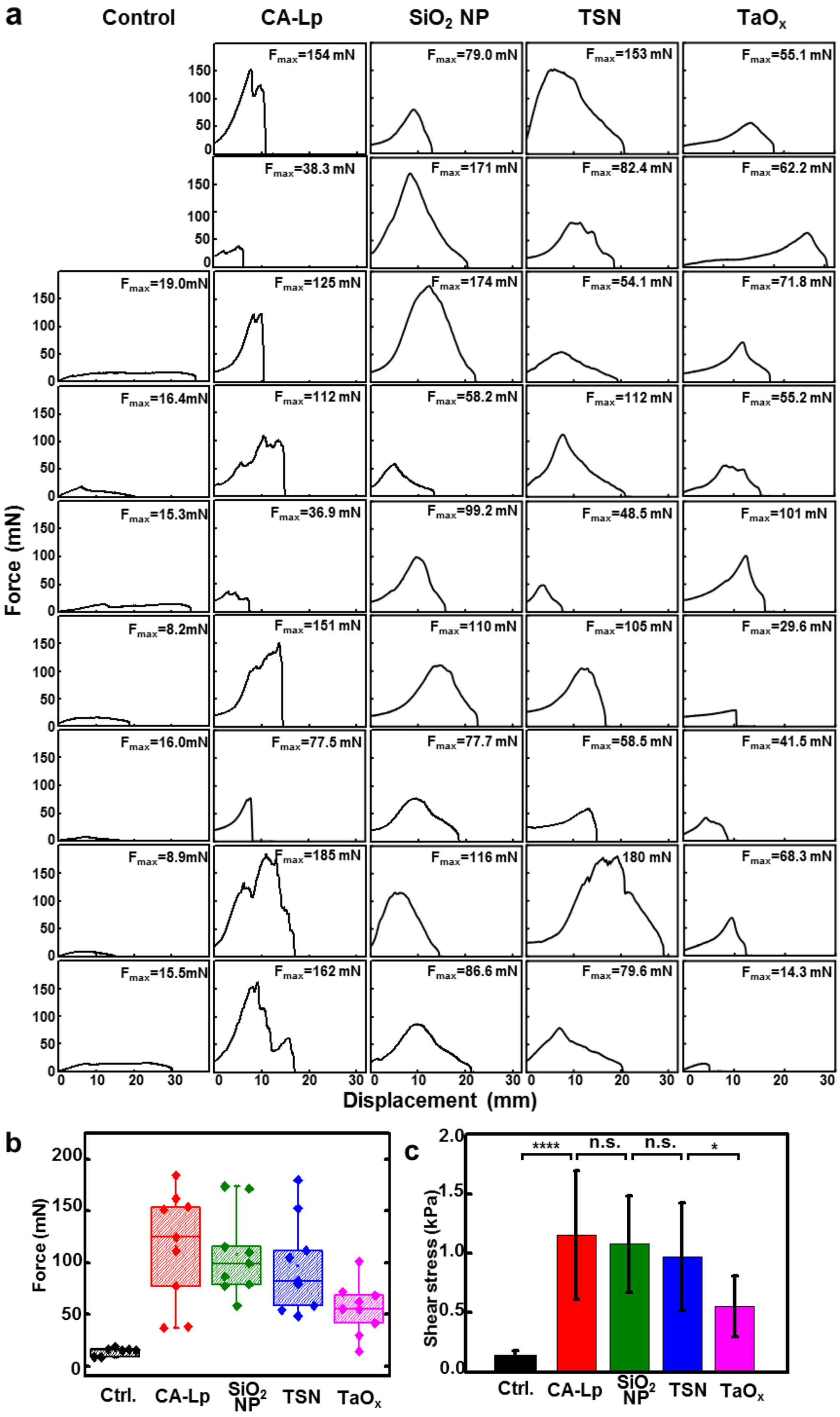

a
Control
CA-Lp
SiO2 NP
TSN
TaOx
Fmax=154 mN
Fmax=79.0 mN
Fmax=153 mN
Fmax=55.1 mN
Fmax=38.3 mN
Fmax=171 mN
Fmax=82.4 mN
Fmax=62.2 mN
Fmax=19.0mN
Fmax=125 mN
Fmax=174 mN
Fmax=54.1 mN
Fmax=71.8 mN
Fmax=16.4mN
Fmax=112 mN
Fmax=58.2 mN
Fmax=112 mN
Fmax=55.2 mN
Fmax=15.3mN
Fmax=36.9 mN
Fmax=99.2 mN
Fmax=48.5 mN
Fmax=101 mN
Fmax=8.2mN
Fmax=151 mN
Fmax=110 mN
Fmax=105 mN
Fmax=29.6 mN
Fmax=16.0mN
Fmax=77.5 mN
Fmax=77.7 mN
Fmax=58.5 mN
Fmax=41.5 mN
Fmax=8.9mN
Fmax=185 mN
Fmax=116 mN
180 mN
Fmax=68.3 mN
Fmax=15.5mN
Fmax=162 mN
Fmax=86.6 mN
Fmax=79.6 mN
Fmax=14.3 mN
Force (mN)
Displacement (mm)
b
Force (mN)
Ctrl.
CA-Lp
SiO2 NP
TSN
TaOx
c
Shear stress (kPa)
****
n.s.
n.s.
*
Ctrl.
CA-Lp
SiO2 NP
TSN
TaOx

Supplementary Figure 5 | Evaluation of the adhesion strengths of the liver ribbon lab joint attached with the different adhesives. a, Normalized force-displacements curves of the lab joints made of ribbons cut from the calf liver and glued with intrinsic adhesive property of biological tissues (Control), CA-Lp, SiO_2 NPs, surfactant-free TaO_x/SiO_2 core/shell nanoparticles (TSN), and TaO_x NPs without SiO_2 shell. The failure force is shown upper left side of each curve. **b,** Box plot for the failure forces of each adhesive. Each failure force is denoted with diamond, bars represent maximum and minimum values, and boxes with line are represented the interquartile range with median. **c,** Bar plot of mean shear stress. Error bars show the standard deviation. *p* values were calculated by one-way ANOVA test. ****$p<0.0005$, *$p<0.05$, n.s., not significant $p>0.05$.

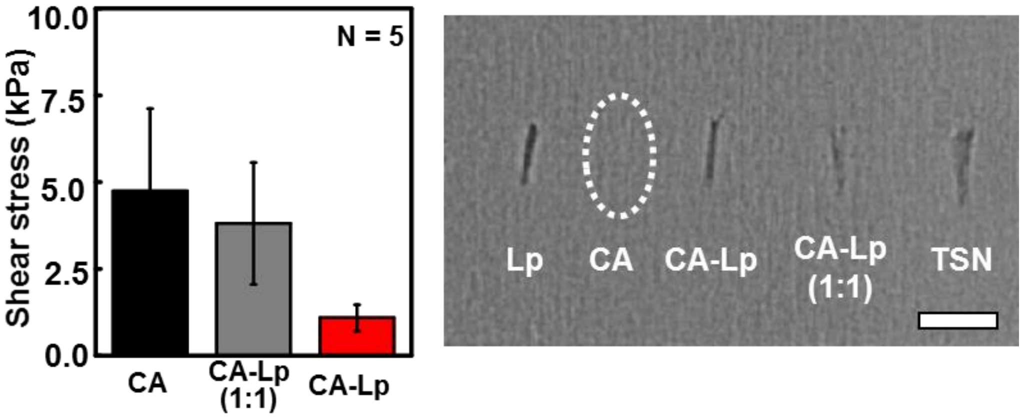

Supplementary Figure 6 | Comparison of CA and CA-Lp mixture. a, Mean shear stress with standard deviation as error bars of cyanoacrylate (CA; Histoacryl) and cyanoacrylate-Lipiodol mixture (CA-Lp) with a volume ratio of 1:1 and 1:3. **b,** Fluoroscopy image of applied adhesives in agarose gel. White dashed circle indicates the position where CA was applied. Scale bar indicates 1 cm.

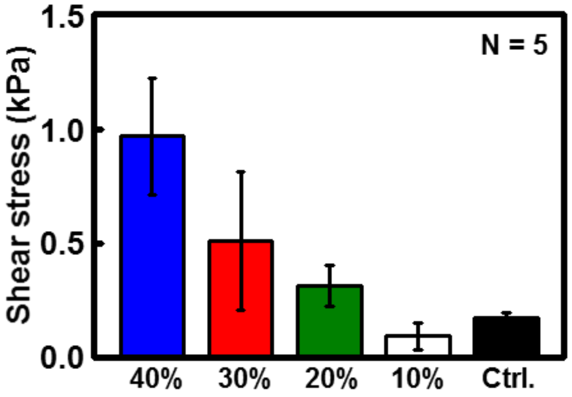

Supplementary Figure 7 | Concentration dependency of the adhesive strength of TSNs. Mean shear stress with standard deviation as error bars of TSNs depending on the concentrations of the solution including 40%, 30%, 20%, and 10% in weight percentages. Control group (Ctrl.) is intrinsic adhesion of the ribbons of calf liver.

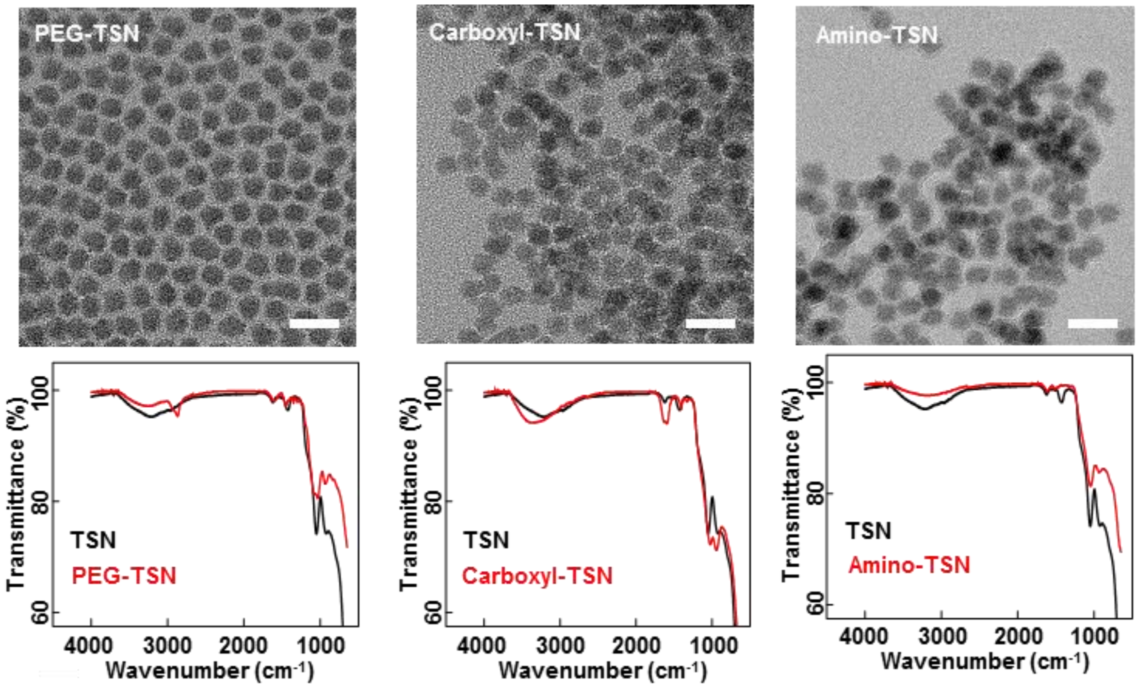

Supplementary Figure 8 | TEM images and FT-IR spectra TSNs with modified surfaces. Transmission electron images with surface modified tantalum oxide/silica core/shell nanoparticles having various moieties on the surface including polyethylene glycol (PEG-TSN), carboxylate (Carboxyl-TSN), and primary amine (Amino-TSN). Scale bars indicate 20 nm. Fourier transform infrared spectra of each modified TSNs (red) and TSN with bare silica surface (black).

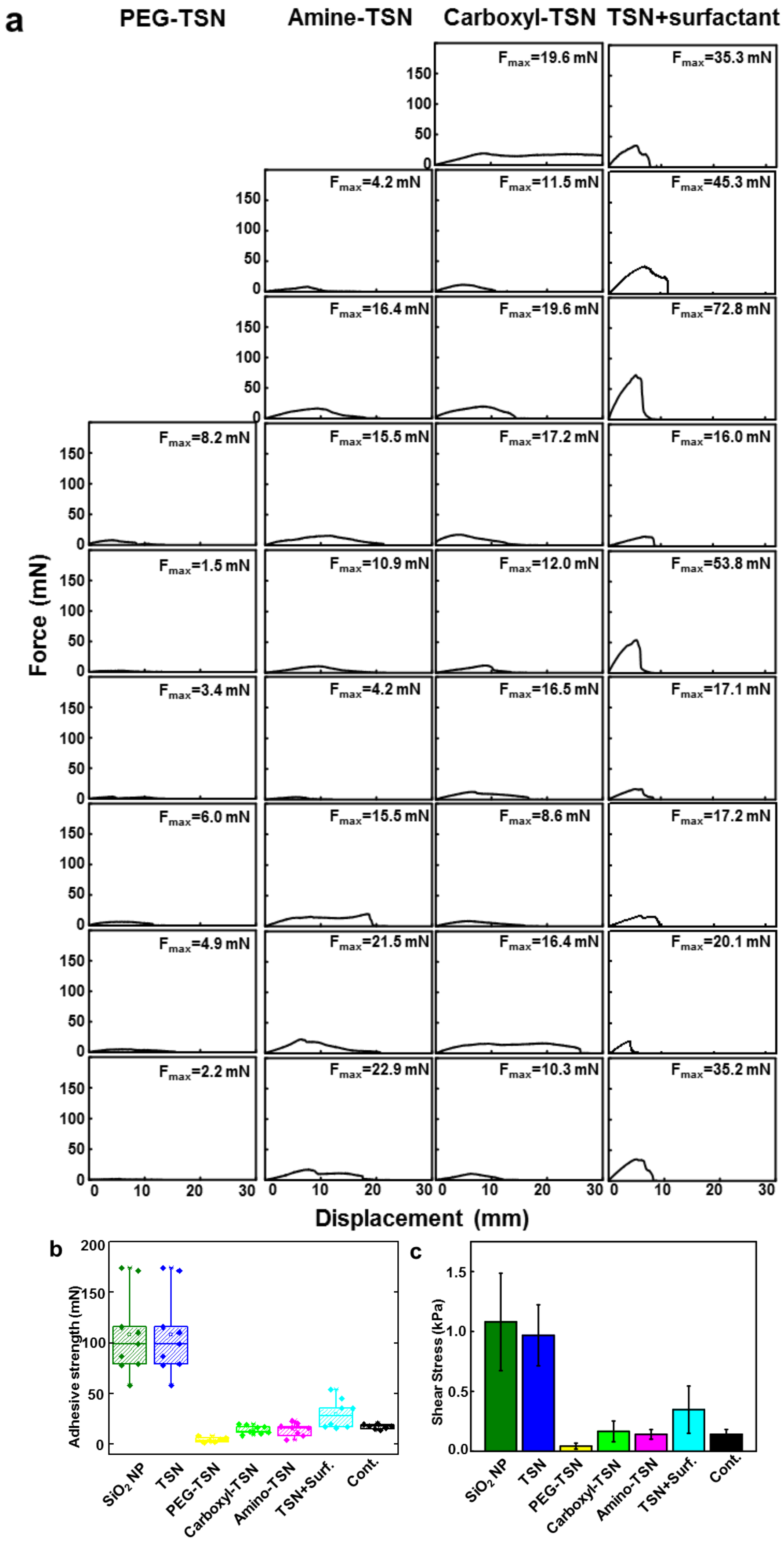
a
PEG-TSN
Amine-TSN
Carboxyl-TSN
TSN+surfactant
Fmax=19.6 mN
Fmax=35.3 mN
Fmax=4.2 mN
Fmax=11.5 mN
Fmax=45.3 mN
Fmax=16.4 mN
Fmax=19.6 mN
Fmax=72.8 mN
Fmax=8.2 mN
Fmax=15.5 mN
Fmax=17.2 mN
Fmax=16.0 mN
Fmax=1.5 mN
Fmax=10.9 mN
Fmax=12.0 mN
Fmax=53.8 mN
Fmax=3.4 mN
Fmax=4.2 mN
Fmax=16.5 mN
Fmax=17.1 mN
Fmax=6.0 mN
Fmax=15.5 mN
Fmax=8.6 mN
Fmax=17.2 mN
Fmax=4.9 mN
Fmax=21.5 mN
Fmax=16.4 mN
Fmax=20.1 mN
Fmax=2.2 mN
Fmax=22.9 mN
Fmax=10.3 mN
Fmax=35.2 mN
Force (mN)
Displacement (mm)
b
Adhesive strength (mN)
c
Shear Stress (kPa)
SiO2 NP
TSN
PEG-TSN
Carboxyl-TSN
Amino-TSN
TSN+Surf.
Cont.

Supplementary Figure 9 | Adhesion strengths of various nanoparticles. a, Normalized force-displacements curves of PEG-TSNs, Amine-TSNs, Carboxyl-TSNs, surfactant-adsorbed TSNs (TSN+Surfactant; without acid treatment). The failure force value is shown upper left side of each curve. **b,** Box plot for the failure force of each adhesive. Each failure force is denoted with diamond. Bars represent maximum and minimum values. Boxes with line represent the interquartile range with median. **c,** Bar plot of mean shear stress. Error bars show the standard deviation.

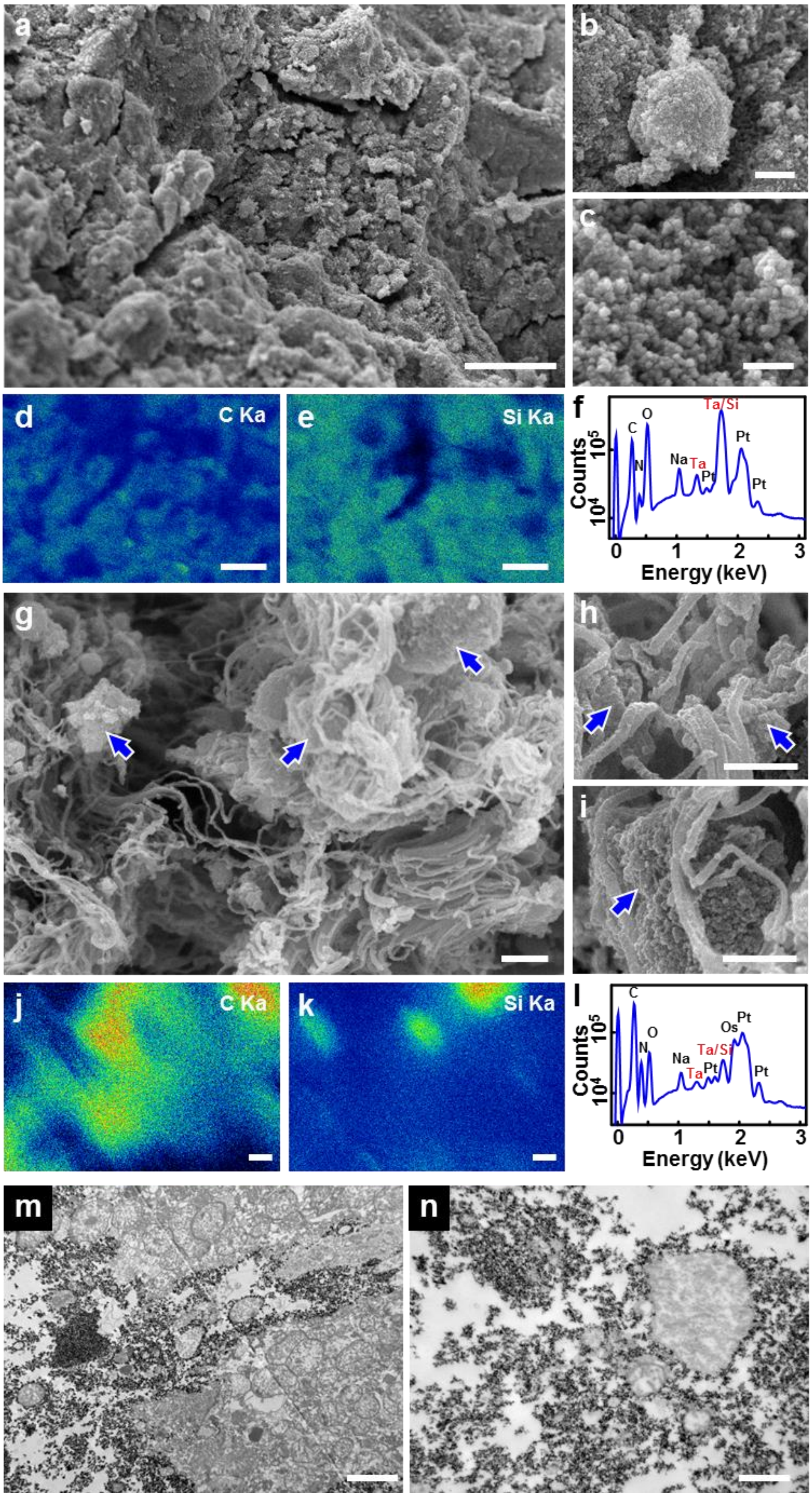
a
b
c
d
C Ka
e
Si Ka
f
Counts
Energy (keV)
C
N
O
Na
Ta
Pt
Ta/Si
Pt
Pt
g
h
i
j
C Ka
k
Si Ka
l
Counts
Energy (keV)
C
N
O
Na
Ta
Pt
Ta/Si
Os
Pt
Pt
m
n

Supplementary Figure 10 | Electron microscopic images of TSN-applied liver tissue. a, Scanning electron microscope(SEM) image of the side of liver where TSNs applied. (Scale bar: 10 μm) **b,** SEM image showing nanoparticles coating the leaked vesicle by adsorption. (Scale bar: 500 nm) **c,** Highly magnified image showing the adsorbed nanoparticles. (Scale bar: 100 nm) **d-e,** Carbon and silicon elemental mapping of the image (a) by energy-dispersive X-ray spectroscopy (EDS) showing wide adsorption of TSN onto the liver section. (Scale bar: 10 μm) **f,** EDS spectra of image (a) showing the tantalum and silicon peaks. **g,** SEM image where fibrin fibers are networked with TSN. The blue arrows point the locations of TSN agglomerates. (Scale bar: 1 μm) **h-i,** Highly magnified images in figure (g) showing the adsorption of TSN near fibrin fiber (h) and entanglement of TSN agglomerates within the fibrin fiber. (Scale bar: 500 nm) **j-k,** Carbon and silicon elemental mapping of the image (g) by EDS. (Scale bar: 1 μm) **l,** EDS spectra of image (g). **m-n,** Transmission electron microscope (TEM) images showing the adsorption of the TSN near and between hepatocytes (m; scale bar is 2 μm) and leaked vesicles (n; scale bar is 500 nm) from the damaged hepatocytes.

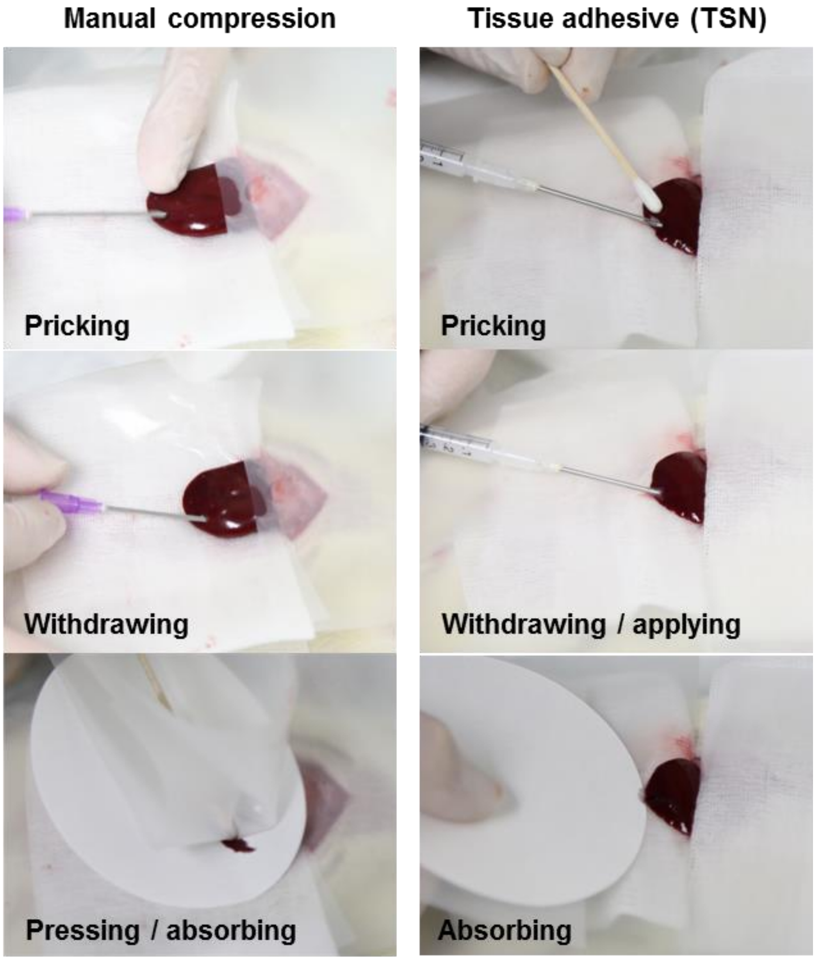

Supplementary Figure 11 | Procedures of evaluating hemostatic effects. After abdominal incision, the left lobe of the liver was placed on a hydrophobic film (Parafilm). An 18-gauge needle was advanced into the liver. Immediately after withdrawing the needle, prepared cotton swabs covered with a Parafilm and a filter paper were put onto the wound and manual compression was applied. On the other hand, prepared TSN or CA-Lp was injected during the withdrawal of the needle, and bleeding or residual adhesives were absorbed with a filter paper.

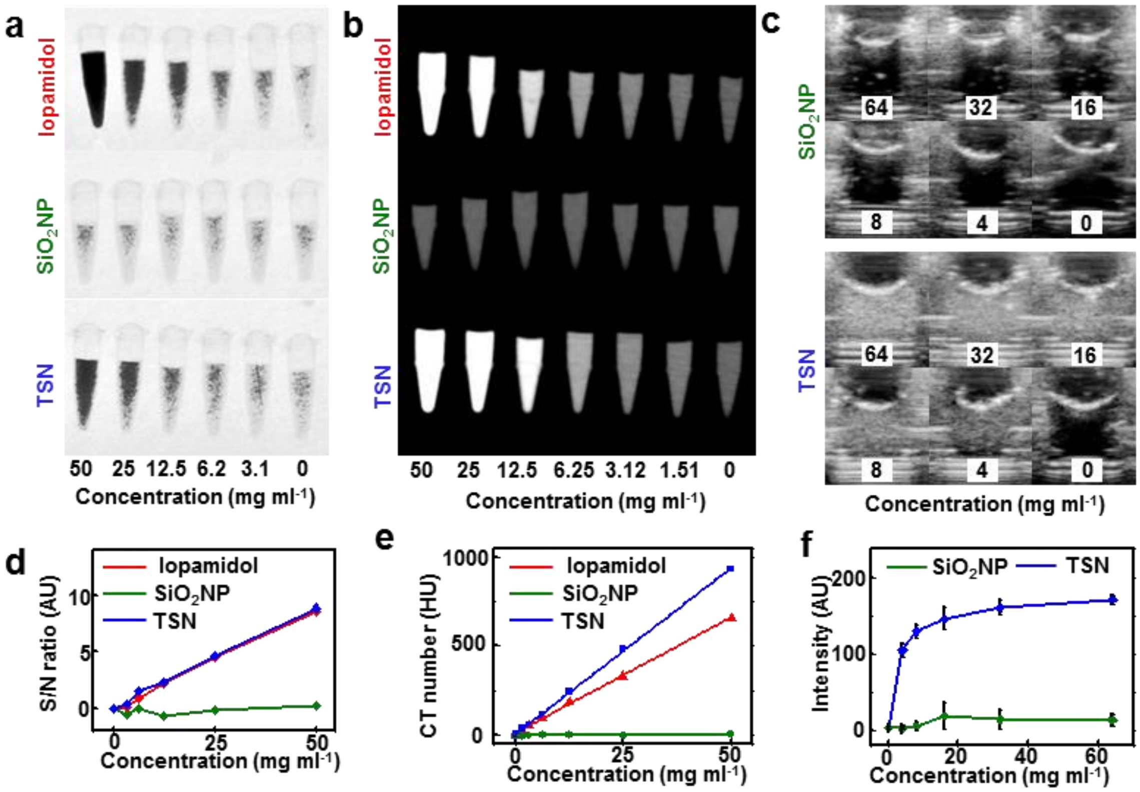

Supplementary Figure 12 | Comparison of the contrast effects. a, X-ray Fluoroscopic images of the microtubes containing Iopamidol, SiO_2 NPs, TSNs with various concentrations. **b,** CT images of the microtubes containing the Iopamidol, SiO_2 NPs, and TSNs. **c,** Ultrasound images of the agarose phantom containing NPs with various concentration. **d,** Plots of the signal-to-noise ratio increase depending on the concentration of the Iopamidol (red), SiO_2 NPs (green), and TSNs (blue) calculated on fluoroscopic images (a). **e,** Plots of the CT values of the Iopamidol (red), SiO_2 NP (green), TSN (blue) calculated from CT images (b). **f,** Plots of the signal intensity increase of the SiO_2 NPs (green), TSNs (blue) from ultrasonography (c).

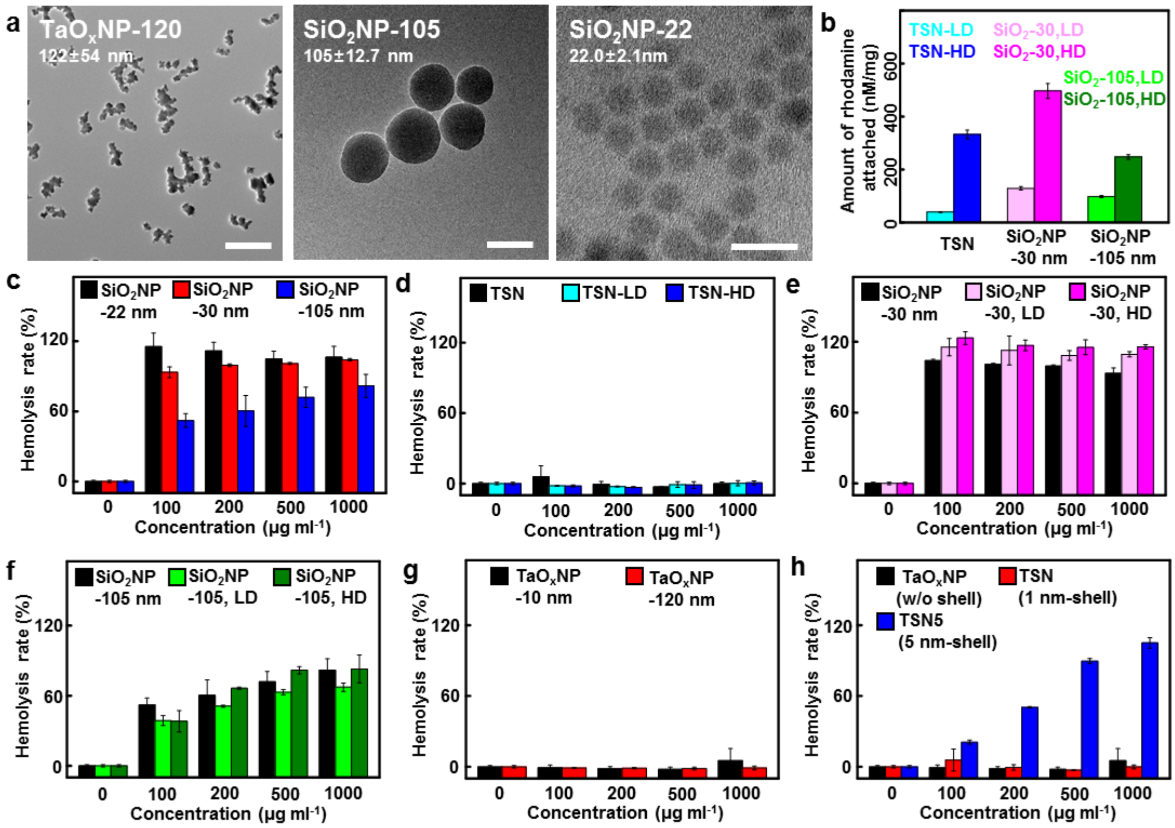

Supplementary Figure 13 | Comparative study of hemolysis effects. a, Transmission electron microscope (TEM) images of TaO_x NPs-120 nm (scale bar is 500 nm), SiO_2 NPs-105 nm (Stober silica; scale bar is 100 nm), and SiO_2 NPs-22 (scale bar is 100 nm) additionally prepared for hemolysis evaluation. **b,** Quantification of rhodamine attached onto the tantalum oxide/silica core/shell nanoparticle (TSN), SiO_2 NP-30 (Ludox TM-50; TEM image in Supplementary Figure 4d), and SiO_2 NP-105, prepared with low (LD) and high (HD) dye concentration. **c,** Hemolysis rate of silica nanoparticles with sizes of 22 nm, 30 nm, and 105 nm reveals that the largest 105 nm-sized NPs exhibit lower hemolysis rate. **d-f,** Hemolysis rates of TSNs, SiO_2 NP-105, and SiO_2 NP-30 with different amount of rhodamine dye incorporation reveal that the amount of incorporated dye does not affect the hemolysis rate. **g,** Hemolysis rate of TaO_x NPs with sizes of 11 nm and 120 nm reveals that both TaO_x NPs exhibit negligible hemolysis effect. **h,** TSNs with thicker silica shell exhibits higher hemolysis rate than TSNs with thinner silica shell and TaO_x NPs. (Each bar represents mean value, error bar is s.d., n=3)

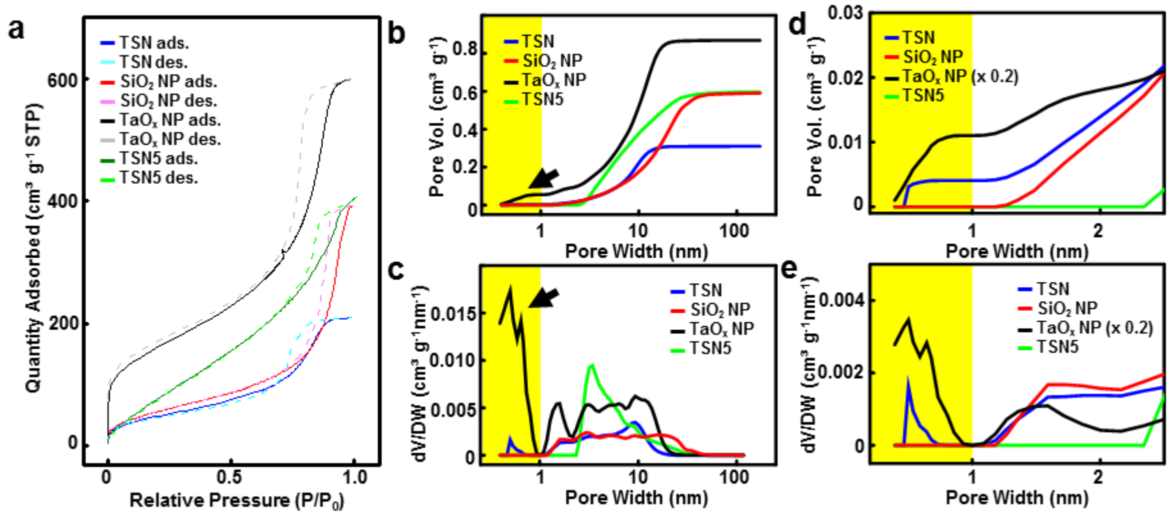

Supplementary Figure 14 | Gas adsorption analysis of various TSNs and SiO_2 NP. a, N_2 adsorption-desorption isotherm of TaO_x/SiO_2 NPs with 1 nm-thick silica shell (denoted as TSN), SiO_2 NP, TaO_x NP, TaO_x/SiO_2 NPs with 5 nm-thick silica shell (denoted as TSN5). **b-c,** Cumulative pore volume and pore size distributions. The black arrows indicate the microporous structure of TaO_x NPs. **d-e,** enlarged plots showing the micro-pore region. The mirco-pore region is shaded with yellow box. Pore size distributions were calculated with density functional theory method.

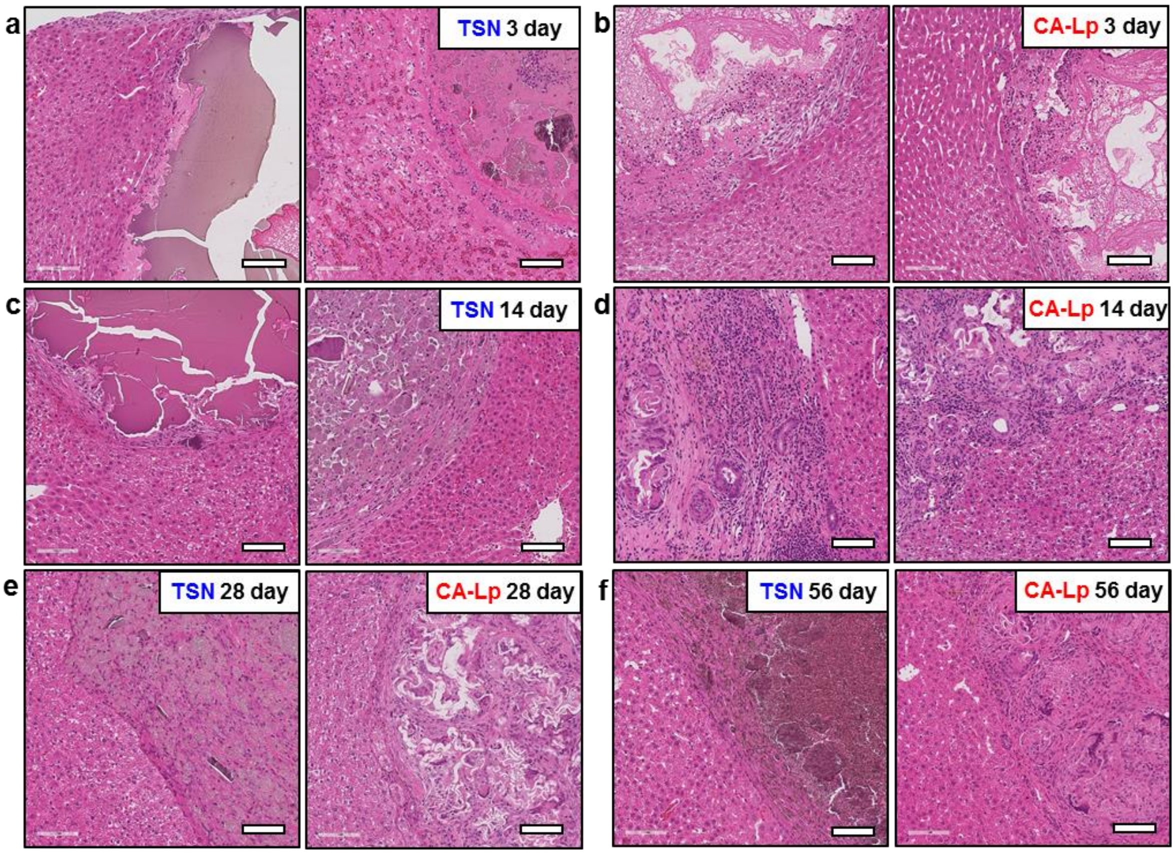

Supplementary Figure 15 | Histological analyses for biocompatibility of TSN compared to CA-Lp mixture applied in the liver. Tissue specimens stained with hematoxylin and eosin (H&E) 3 days after implantation of **(a)** TSN or **(b)** CA-Lp, 14 days after implantation of **(c)** TSN or **(d)** CA-Lp, **(e)** 28, and **(f)** 56 days after the implantation. Note that immune response rarely occurred after TSN implantation, while numerous immune cells were recruited after CA-Lp implantation. The scale bars indicate 100 μm.

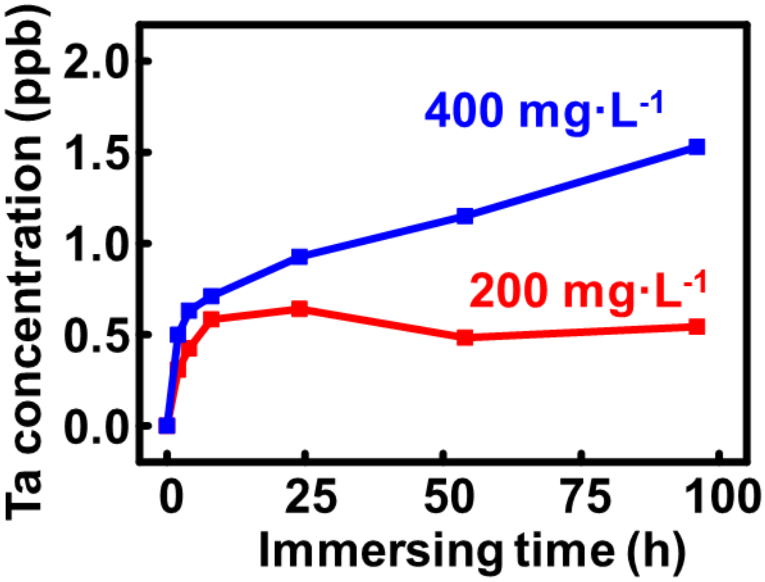

Supplementary Figure 16 | Dissolution profile of tantalum element from TSNs. The concentration of dissolved Ta from TSNs in simulated body fluid, depending on immersing time and immersed amount of the TSNs.

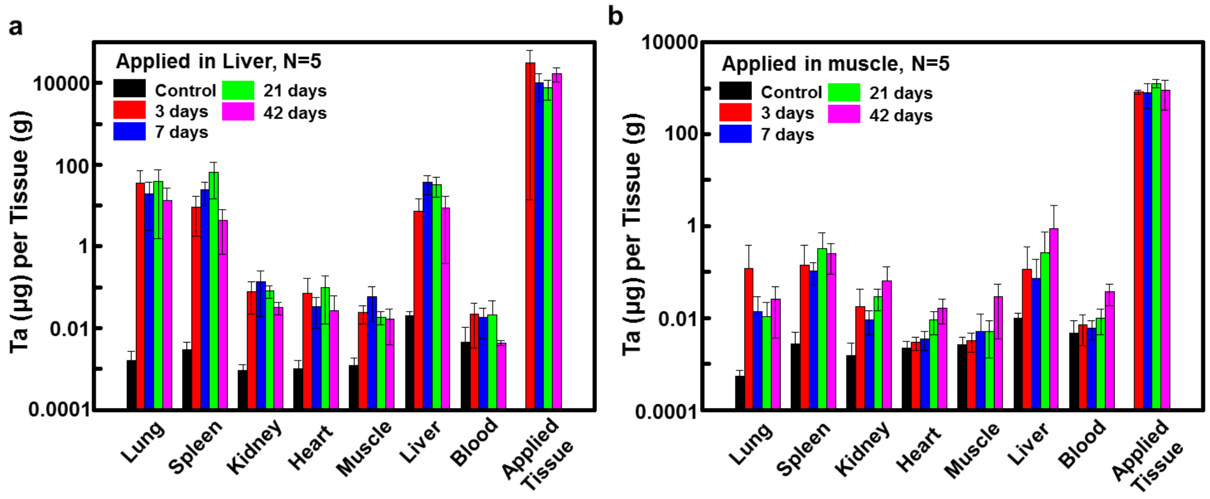

Supplementary Figure 17 | *In vivo* distribution of Ta element from TSNs. Average concentration of tantalum element in tissues obtained at 3, 7, 21, and 42 days after the application of TSNs as hemostatic agent for liver puncture models (a), and as fiducial marker for the muscles (b).

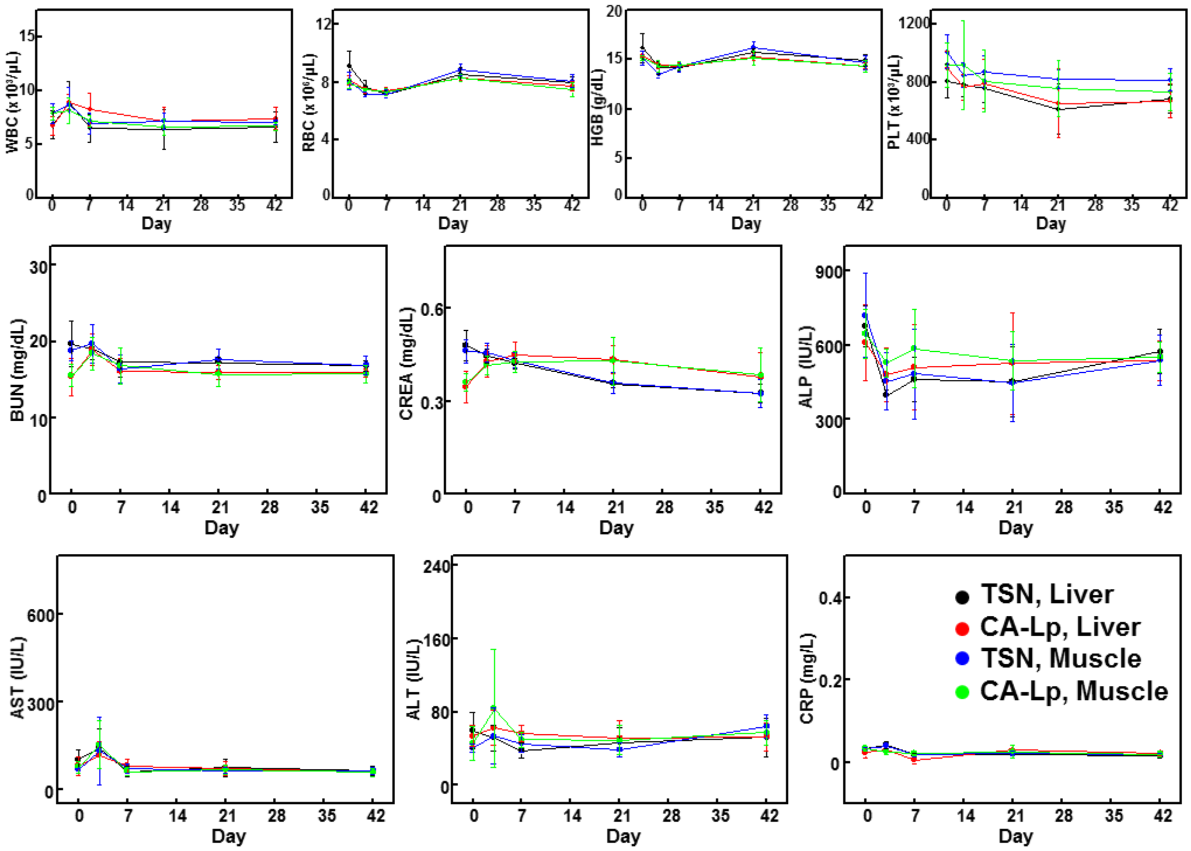

Supplementary Figure 18 | Blood chemistry analyses for in vivo biocompatibility study. Long-term complete blood count (CBC) results for WBC, RBC, HGB, and PLT. Long-term serum biochemistry results for BUN, CREA, ALP, AST, ALT, and CRP from the Sprague Dawley rats. The analyses were proceeded after applying glues (TaO_x/SiO_2 nanoparticles (TSNs), mixture of cyanoacrylate and Lipiodol (CA-Lp)) to the liver and muscle. (Data are shown as mean ± standard deviation, n = 5.)

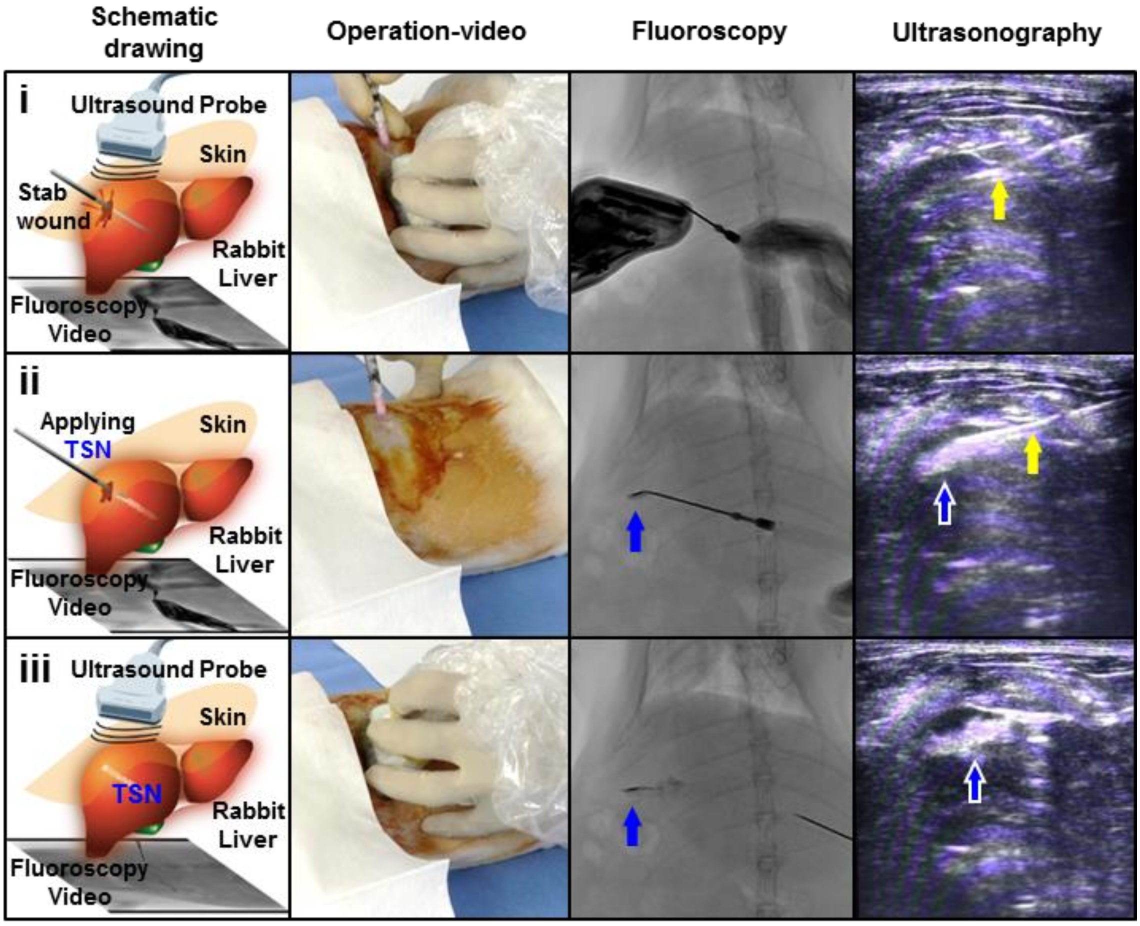

Supplementary Figure 19 | Demonstration of image guided procedure using TSNs. Series of schematic drawings, still images of operation-video, real-time fluoroscopic video, and ultrasonography. Each step represents; (**i**) making a stab wound with a needle, (**ii**) applying TSNs for hemostasis during withdrawal of the needle, and (**iii**) imaging for the post-procedural validation. On the images, blue and yellow arrows indicate tantalum oxide/silica core/shell nanoparticles (TSNs) and a needle, respectively.

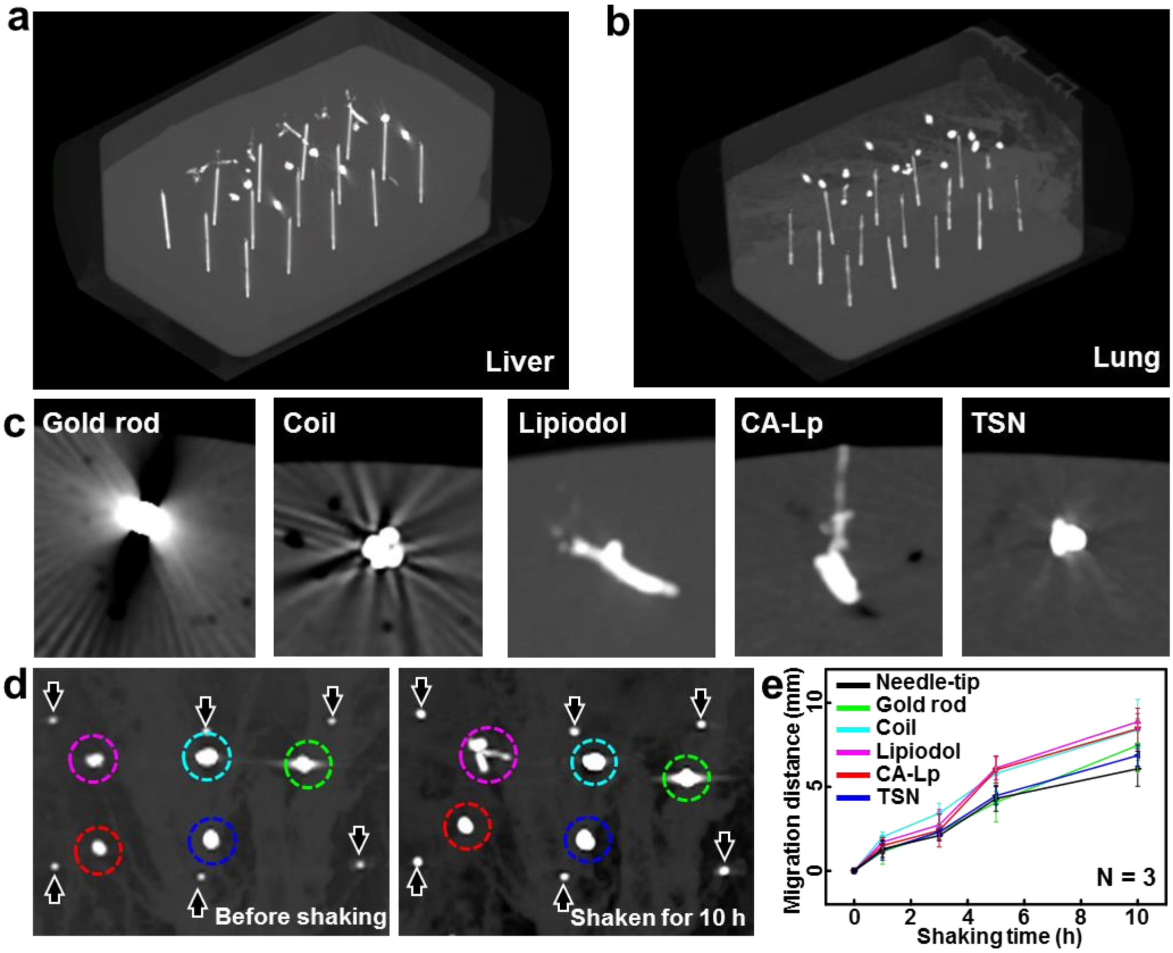

Supplementary Figure 20 | Mobility evaluation of fiducial markers. a-b, three-dimension rendered X-ray CT images of the liver (a) and lung (b) which contains various fiducial markers including tantalum oxide/silica core/shell nanoparticles (TSNs), gold rod, metal coil, Lipiodol, and mixture of cyanoacrylate and Lipiodol (CA-Lp). **c**, CT images of each marker. The streak artifacts around the gold rod and coil hinder accurate imaging of the nearby tissue. **d,** Top views of the lung showing the positions of the fiducial markers and needles, before shaking and after 10 hour shaking. Black arrows indicate needle tips, and dashed circles indicate the markers such as TSNs (blue), gold rod (green), metal coil (cyan), Lipiodol (magenta), and CA-Lp (red). **e,** Migration distances of markers in the lung. Data are shown as mean ± standard deviation, n = 3.

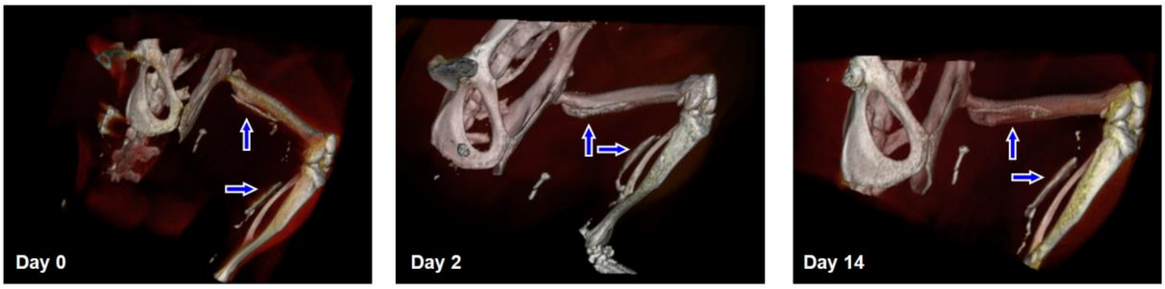

Supplementary Figure 21 | 3D-rendered micro-CT images of TSN-marked leg muscles. Images were obtained right after the implementation, after 2 days passed, and after 2 weeks passed. The two blue arrows indicate the TSNs markings in the thigh and calf muscles along with the fibula and femur.

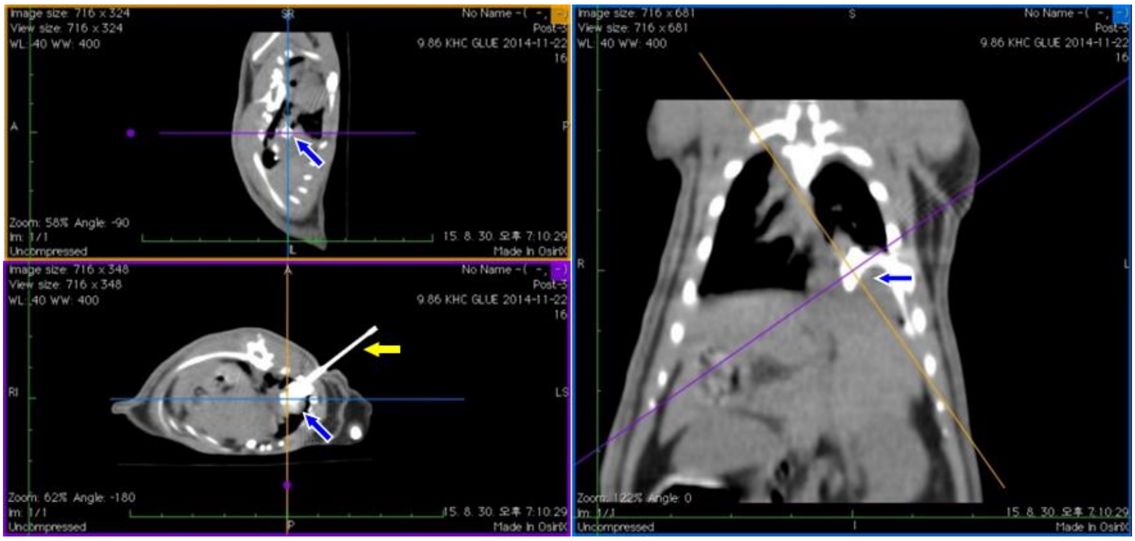

Supplementary Figure 22 | Procedure for marking lung cancer with TSNs. Multiplanar-reformatted CT images during percutaneous lung tumor marking procedure with fluorescent TSNs. The needle and injected tantalum oxide/silica core/shell nanoparticles (TSNs) are visualized on an axial cross-section image. Blue and yellow arrow indicate injected TSNs and the needle, respectively.

Sample	Trialkoxysilane for surface modification	Reaction condition		Zeta-potential (mV)	Hydrodynamic diameter (nm)
		Solvent	T(°C)		
Bare-TSN	None			-41.9 ± 4.1	20.4±7.2
PEG-TSN	2-[methoxy (polyethyleneoxy) 21-24propyl] trimethoxysilane (Tech-90, Gelest)	Following previous reports[1]		-32.3± 9.0	17.0±5.2
Carboxyl-TSN	N-(trimethoxysilyl propyl) ethylenediamine triacetate (35% in water, Gelest)	Water	25	-36.6± 10.6	18.4±5.3
Amino-TSN	3-aminopropyltriethoxysilane (98%, Sigma-Aldrich)	Ethanol	70	38.0± 5.7	64.1±21.8

Supplementary Table 1. preparation method and characterization of modified TSNs. Summary of reagents for grafting surface, preparation condition, zeta-potentials and hydrodynamic diameters of various surface modified TSNs.

Supplementary References

1. Oh, M. H. *et al.* Large-scale synthesis of bioinert tantalum oxide nanoparticles for x-ray computed tomography imaging and bimodal image-guided sentinel lymph node mapping. *J. Am. Chem. Soc.* **133**, 5508-5515 (2011).
